# Climatically robust multiscale species distribution models to support pronghorn recovery in California

**DOI:** 10.1002/ece3.11454

**Published:** 2024-06-20

**Authors:** William T. Bean, H. Scott Butterfield, Jeanette K. Howard, Thomas J. Batter

**Affiliations:** ^1^ California Polytechnic State University – San Luis Obispo San Luis Obispo California USA; ^2^ The Nature Conservancy Sacramento California USA; ^3^ New Mexico Department of Game and Fish Santa Fe New Mexico USA

**Keywords:** *Antilocapra americana*, arid grassland, climate change, habitat, migration, niche reduction, ungulate

## Abstract

We combined two climate‐based distribution models with three finer‐scale suitability models to identify habitat for pronghorn recovery in California now and into the future. We used a consensus approach to identify areas of suitable climate now and future for pronghorn in California. We compared the results of climate models from two separate hypotheses about their historical ecology in the state. Under the migration hypothesis, pronghorn were expected to be limited climatically by extreme cold in winter and extreme heat in summer; under the niche reduction hypothesis, historical pronghorn of distribution would have better represented the climatic limitations of the species. We combined occurrences from GPS collars distributed across three populations of pronghorn in the state to create three distinct habitat suitability models: (1) an ensemble model using random forests, Maxent, classification and regression Trees, and a generalized linear model; (2) a step selection function; and (3) an expert‐driven model. We evaluated consensus among both the climate models and the suitability models to prioritize areas for, and evaluate the prospects of, pronghorn recovery. Climate suitability for pronghorn in the future depends heavily on model assumptions. Under the migration hypothesis, our model predicted that there will be no suitable climate in California in the future. Under the niche reduction hypothesis, by contrast, suitable climate will expand. Habitat suitability also depended on the methods used, but areas of consensus among all three models exist in large patches throughout the state. Identifying habitat for a species which has undergone extreme range collapse, and which has very fine scale habitat needs, presents novel challenges for spatial ecologists. Our multimethod, multihypothesis approach can allow habitat modelers to identify areas of consensus and, perhaps more importantly, fill critical knowledge gaps that could resolve disagreements among the models. For pronghorn, a better understanding of their upper thermal tolerances and whether historical populations migrated will be crucial to their potential recovery in California and throughout the arid Southwest.

## Graphical abstract

Identifying habitat for a population no longer occupying large swaths of historical areas poses a number of problems for ecologists. We used a suite of modeling approaches to compare estimates of contemporary and future habitat for pronghorn in California. Large areas will remain suitable for the species, but manipulation of local habitat for increased movement and water availability, and a better understanding of historical migration patterns are needed.

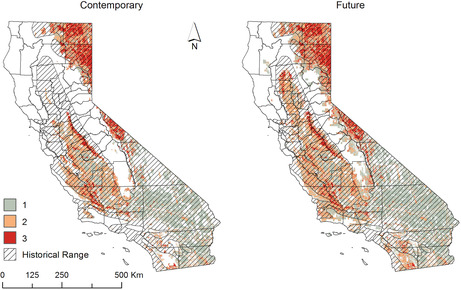

## 1 INTRODUCTION

Pronghorn (*Antilocapra americana*), the sole extant member of family Antilocapridae, play a foundational ecological and cultural role in the ecosystems of western North America, including California (Bean et al., 2024; O'Gara & McCabe, 2004). The species is broadly distributed throughout arid parts of the continent from northern Mexico to southern Canada (Yoakum, 2004a). Total population of pronghorn is estimated to have once numbered in the tens of millions, but by the 1920s due to unregulated harvest they decreased to fewer than 30,000 (Yoakum, 2004a). Subsequent conservation initiatives have facilitated the recovery of populations in the Great Basin, Rocky Mountains, and Great Plains. Over the past two decades, the total population has stabilized around 1 million (Norton & Lindbloom, in press; Yoakum, 2004a). Recovery in California, however, has not matched that observed in other regions (Batter, 2023; Sommer, 2012).

Pronghorn in California are separated into two distinct ecological regions: southern California and the Great Basin populations (Bean et al., 2024). Early European records point to widespread herds at high densities, notably in the Great Central Valley of California (Brown et al., 2006). By the 1920s, Nelson (1925, cited in Brown, 2006) estimated that just 1070 pronghorn remained in the state, with over 90% found on the Modoc Plateau (part of the Great Basin populations) and smaller, fragmented populations in southern California regions like the Mojave and Colorado deserts. The northeastern Great Basin population in California has recovered to the point where regulated harvest has been an annual element since 1964, although managers have observed periodic declines often following harsh winters, and there is some concern about recent declines tied to reduction in habitat quality (Sommer, 2012). Managers have periodically attempted to translocate pronghorn from Modoc and Lassen Counties into historical vacant range throughout southern California (CDFG, 1990; Sommer, 2012). Although small populations have been restored, these efforts have had limited success. Each of the reintroduced populations has experienced a decline in abundance (Koch & Yoakum, 2002; Sommer, 2012), and many are once again considered extirpated (J. Cann and T. Kasteen, personal communication). The underlying drivers of these challenges, likely rooted in pronghorns' complex habitat requirements (Bean et al., 2024) remain largely undetermined.

It is hypothesized that California's pronghorn translocation failures are a result of a combination of physiological, phenological, and genetic characteristics of the source populations poorly matching unique environmental characteristics of southern California (Bean et al., 2024). These challenges have been further complicated by a rapidly changing climate that is making pronghorn habitat in drier desert regions overall hotter and drier for longer portions of the year (Gedir et al., 2015; Seager et al., 2007). Pronghorn in desert states, where precipitation is a major driver of population dynamics, experience a summer bottleneck (Bean et al., 2024; Bender et al., 2013; Gedir et al., 2015). There is some evidence that pronghorn in desert states may be limited by access to free water, particularly post‐parturition (Bender & Rosas‐Rosas, 2021; Morgart et al., 2005) and that individuals can suffer hyperthermia in extreme temperatures (Wilson & Krausman, 2008). As water becomes more limiting in these increasingly arid desert regions, the free water that remains is often used heavily by pronghorn and their predators, including coyotes, potentially impacting fawn survival (Rich et al., 2019); access to free water may also result in greater competition with other ungulates, particularly feral horses (Gooch et al., 2017). In addition to natural limitations, pronghorn are highly risk averse, avoiding major roads and other human development (Gavin & Komers, 2006; Reinking et al., 2019). Fences provide major barriers to movement unless modified to support passage (Jones et al., 2018; Robb et al., 2022; Yoakum, 2004b).

Despite these challenges, conservation organizations, including The Nature Conservancy, and federal and state land managers have prioritized the protection, management, and recovery of pronghorn statewide. They have placed a a renewed emphasis on southern California populations, like those found at the Carrizo Plain, which are likely experiencing population bottlenecks that could lead to extirpation. Efforts are underway to assess whether better management of pronghorn habitat—including establishment and maintenance of free‐standing water across occupied habitat, removal or modifying of fences, and restoration of habitat to provide year‐long forage sources—can increase reproduction, fawn survival, and overall abundances. However, it is clear that more work needs to be done to identify and protect potential pronghorn habitat with the long‐term goal to connect pronghorn populations across California and reduce the pressure of genetic bottlenecks (Dunn & Byers, 2008; Hahn & Culver, 2021).

Historically, protection and maintenance of potential pronghorn habitat focused exclusively on previously extirpated habitat together with reintroductions—the natural or assisted movement of individuals into previously extirpated habitat—from stable and growing populations. Increasingly, however, climate suitability is also a major factor in reintroduction success (Bellis et al., 2020). Reintroduction campaigns have traditionally focused on biotic factors at reintroduction sites because the climate was assumed stable over the relevant management time span (Bellis et al., 2021). However, anthropogenic climate change now forces conservation scientists to account not just for contemporary climate suitability but also consider future changes (Gomides et al., 2021).

Species distribution models (SDMs) (Elith & Leathwick, 2009) have become a powerful tool for addressing future climate suitability for wildlife management and recovery efforts, including identifying sites suitable for reintroduction (Osborne & Seddon, 2012). Often, these models are used to locate sites with appropriate biotic habitat components (for example, land cover types or anthropogenic impacts), but researchers have increasingly turned to climate‐driven models to estimate suitability now (Anderson et al., 2009) and into the future (Bellis et al., 2021; Maes et al., 2019; Martínez‐Meyer et al., 2006; Stewart et al., 2019). Correlative SDMs use a variety of statistical techniques to relate occurrence records with environmental covariates (Elith & Leathwick, 2009). This approach makes several key assumptions about a species niche—perhaps the most important being that a species is at equilibrium with its environment (i.e., not undergoing significant range expansion or collapse) (Elith & Leathwick, 2009; Peterson et al., 2011). When modeling landscapes where a species has been extirpated to project future climate suitability, this assumption is violated, as the model is used to identify sites that the species no longer occupies. In this case, a species has undergone a niche reduction, in which anthropogenic impacts have systematically eroded parts of its realized niche (Scheele et al., 2017). Therefore, a model of the contemporary distribution will underestimate, and spatially bias, areas suitable for future climates (Rutrough et al., 2019; Stewart et al., 2019). The difficulty of identifying suitable climatic sites for acquisition/reintroduction/management is further compounded for migratory or nomadic species, where temporally static SDMs ignore the seasonal restrictions that often caused long‐range movements to evolve (Hayes et al., 2015). However, identifying suitable sites should not simply rely on climate for indicators of suitability but also incorporate biotic factors (Osborne & Seddon, 2012).

Finer‐scale habitat suitability modeling using biotic predictors is another commonly used approach for identifying suitable sites for acquisition, reintroduction or management (Hunter‐Ayad et al., 2019). Like SDMs, this approach makes several assumptions about animal habitat selection (Manly et al., 2002) which may be violated by populations in decline (Semerdjian et al., 2021). For example, animals are assumed to make optimal decisions to select habitat components that maximize fitness (Morrison et al., 2006). Declining populations likely do not have high quality habitat available; therefore, the selection decisions identified from a model of these populations would lead to incorrect inference about suitability. Similarly, reintroduced populations often do not have sufficient information with which to make optimal decisions about selection (Farnsworth et al., 2015).

Because of their ecological, cultural and economic importance, and their unique taxonomic lineage, restoration of pronghorn within vacant and suitable habitat is a priority for conservation scientists and wildlife managers in California. Due to the drastic reduction from their historical distribution in California and their migratory or nomadic behavior, the species offers a unique opportunity to develop the use of SDMs to identify sites suitable for acquisition/relocation/management both now and into the future. Suitable land to promote pronghorn population recovery hinges on ecological conditions that can be realistically managed (e.g., fence modification, year‐long artificial water source development, restoration of year‐long forage resources) and those that cannot (e.g., flat areas with suitable climate). Thus, the objectives of this paper are: (1) to identify places in California with suitable climate and topography for pronghorn, now and into the future; and (2) within zones of potentially suitable climate, to identify areas of existing or potential pronghorn habitat to prioritize protection, allow for assisted or natural reintroduction, and provide the foundation for future management.

## 2 METHODS

**FIGURE 1:**
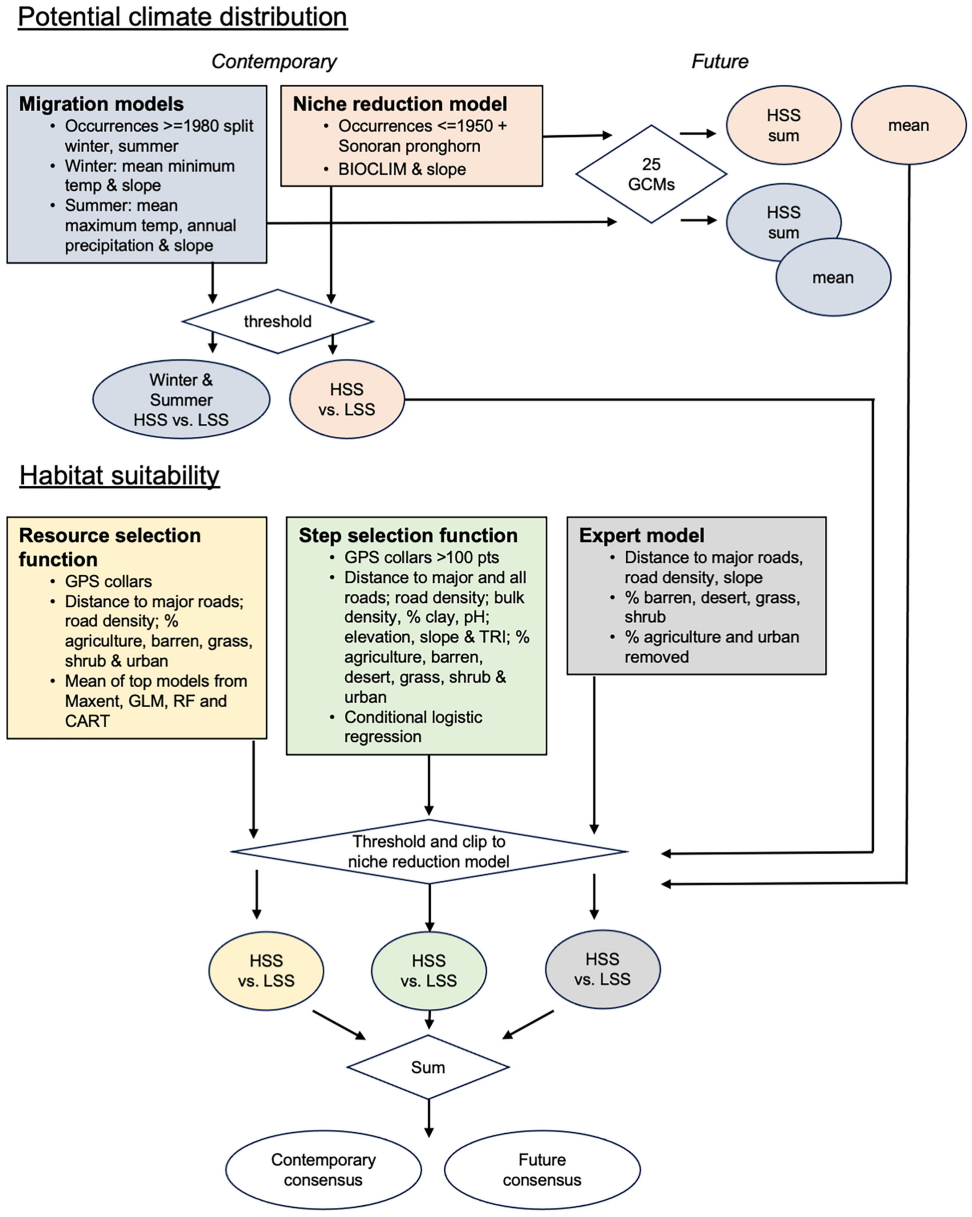
Multiscale, multistep model used to identify pronghorn current and future habitat in California. Models are represented as squares; GIS operations as diamonds; and raster outputs as ovals. We created two climate suitability models (blue and red) based on different assumptions about pronghorn historical ecology and projected both into future climate scenarios. Then, we created three different estimates of habitat suitability (yellow, green, and gray). Each model was thresholded to create an estimate of high (“HSS”) and low (“LSS”) suitability, clipped to the niche reduction model current and future climate; then added together to create a single estimate of consensus for each time period.

We used a multistep, multiscale approach to model pronghorn distribution and habitat in California (Figure 1). First, to estimate the landscape suitability of current and future climate, we devised two distinct sets of species distribution models. The first set of climate‐driven species distribution models (“migration models”) were constructed under the assumption that California pronghorn historically migrated to evade the heat and aridity of summer, and therefore, we used contemporary, range‐wide occurrence data to capture pronghorn movement response to contemporary climate conditions. We modeled pronghorn distribution separately for winter and summer, factoring in season‐specific limiting factors and limiting the model to season‐specific occurrence records. We created the second set of climate‐driven species distribution models (“niche reduction models”) under the premise of the niche reduction hypothesis using only historical records to model pronghorn distribution (Rutrough et al., 2019; Stewart et al., 2019). We projected the top models from both the migration and niche reduction approaches into future climate scenarios.

Then, we modeled contemporary pronghorn habitat in three ways. We used an ensemble modeling strategy (Araújo & New, 2007), leveraging available GPS collar movement data from existing pronghorn populations; we constructed a step selection function (Thurfjell et al., 2014) using the same collar data; and we curated an expert‐driven habitat model (Yamada et al., 2003). Finally, we converted the niche reduction models to a binary suitable or not suitable raster using a threshold value, then used this to clip the habitat models to identify areas of the state with suitable climate and habitat now and into the future.

### 2.1 Potential climate distribution

We acquired pronghorn occurrences from throughout their range from the Global Biodiversity Information Facility (“GBIF”) on April 19, 2023, using the *dismo* package (Version 1.3‐9; Hijmans et al., 2022) in R (Version 4.2.3; R Core Team, 2023). We removed fossil specimens—that no longer represented potential pronghorn habitat—and spatial outliers east of longitude −90°.

We proceeded to construct a set of models of summer and winter climate suitability based on the assumption that pronghorn in California exhibited migratory behavior. Occurrence records were restricted to ≥1980, with spatial precision ≤500 m. We modeled pronghorn distribution for winter (spanning November through February) and summer (from May to September) separately. That is, occurrence data were drawn only from the relevant season and predictor variables were estimated for the relevant month of the occurrence data. The months of March, April and October were excluded, as pronghorn typically migrate during these periods (Collins, 2016).

Winter migration in pronghorn is proximally triggered by cold temperatures and snowpack (e.g., Jakes et al., 2018), while historical summer migrations are hypothesize to have been triggered by heat and need for water (Bean et al., 2024). Therefore, we used only a small number of environmental predictors to capture these necessary migratory conditions for pronghorn. We did this in an attempt to capture the physiological restrictions to winter and summer distribution without incorporating proxies for biotic interactions but used a more traditional approach with a full suite of predictors under the niche reduction hypothesis. For the winter model, predictors included monthly mean minimum temperature and slope. The monthly mean minimum temperature was derived from 30‐year averages (1970–2000) from Worldclim 2.1 (Fick & Hijmans, 2017), recorded at 30‐s resolution, corresponding to the month of each pronghorn occurrence. The slope was determined using elevation data sourced from the NASA Shuttle Radar Topography Mission (2013), acquired via the *dismo* package (Hijmans et al., 2022) in R. The terrain function in the *raster* package (Version 3.6‐20; Hijmans, 2023) in R was used to compute slope. The summer distribution model incorporated mean monthly maximum temperature, slope, and total annual precipitation, with data sourced identically to the winter model.

Next, we constructed a separate set of climate models based on the niche reduction hypothesis. We sought to estimate their historical climatic niche by modeling distribution of pronghorn using historical location data (≤1950). However, in contrast with the American and peninsular subspecies, nearly all records of Sonoran pronghorn from GBIF were from post‐1950. Sonoran pronghorn are a subspecies whose climatic niche aligns more closely with that of pronghorn in southern California (Bean et al., 2024) and therefore can provide important information about the climatic breadth of the species. To address this gap, we included 20 randomly selected locations, representing 10% of the total occurrence records in the model, for Sonoran pronghorn collected after 1950 to supplement the historical occurrence data. We used all 19 bioclimatic variables (representing summaries of monthly, quarterly and annual temperature and precipitation mean, range, maxima and minima) from Worldclim 2.1 and slope as predictors (Fick & Hijmans, 2017).

For both the contemporary migration models and the niche reduction models, we executed a series of models using the ENMEvaluate function with the maxnet algorithm from the *ENMeval* package (Version 2.0.4; Kass et al., 2021) in R. The ENMEvaluate function implements the Maximum Entropy approach to species distribution modeling (Phillips & Dudík, 2008), fitting the most uniform distributions relating relative probability of occurrence to the environmental predictors, subject to observed constraints (Elith et al., 2011). To avoid overfitting, we adjusted the regularization multiplier from 0.5 to 3.0, at 0.5 intervals, and selected the model with the lowest AIC score (Warren & Seifert, 2011). Model evaluations were conducted using five‐fold cross validation, partitioned through the “checkerboard1” setting. To identify the potential climate distribution, we generated a high suitability score (HSS) and low suitability score (LSS) estimate for the migratory and niche reduction models by setting a threshold, and we confined potentially suitable climates to those regions with suitability scores surpassing this threshold. While numerous methods exist for threshold determination with Maxent (Bean et al., 2012), the paucity of historical pronghorn occurrence data in California guided us to choose a threshold that yielded a distribution closely mirroring the most accurate historical pronghorn distribution estimate in California (Brown et al., 2006; Pyshora, 1987).

We extended the winter, summer, and historical models to account for future climate scenarios. Drawing from Worldclim 2.1, we used 25 global circulation models (“gcm”) available for 2041–2060 with representative concentration pathway (“rcp”) 8.5 (Governor's Office of Planning and Research, 2018). This was achieved using the cmip6_world function from the *geodata* package (Version 0.5‐8; Hijmans et al., 2023) in R. Each model was then individually projected onto each gcm. Subsequently, we derived the mean suitability across all circulation models. For each future scenario, we also classified the data into a single suitable/unsuitable scale based on the previously determined thresholds. We then combined the results to procure a secondary consensus estimate.

We evaluated to what extent the future climate projections matched contemporary climate space (Owens et al., 2013). We compared the contemporary climate predictors to the future climate layers using the mobility‐oriented parity (“MOP”) metric (Owens et al., 2013). The MOP metric estimates similarity between two sets of climate data, constrained to areas closer in climate space. The metric ranges from 0 to 1, with higher values representing higher similarity between the contemporary and future climate conditions. We used the kuenm_mop function in package kuenm (Cobos et al., 2019) to calculate the MOP within 10% of the calibration region using 2000 rows for each comparison; because of computing limitations, we limited our analysis to the ACCESS‐CM2 climate projection.

In the context of this study, we gave more credence to the niche reduction hypothesis. This was based on evidence suggesting that pronghorn can adapt to climates more extreme than those currently present in California, as evidenced by populations like the Sonoran and Peninsular pronghorn (Yoakum, 2004a). Conversely, we have not come across documented evidence supporting historical migratory or nomadic behaviors among pronghorn in southern California. As a result, we relied on the findings from the niche reduction models to shape our habitat models but present the migration models to illustrate the importance of understanding the historical migratory or nomadic behavior of the species.

### 2.2 Habitat suitability

We used 2 independent data sources for modeling pronghorn habitat in California: GPS collar data from 3 independent populations within the state, and observations curated and recorded by the California Department of Fish and Wildlife (CDFW). The three sets of GPS collar data were collected independently. One population, in the Bodie Hills region of eastern California, were captured between March 2014 and February 2015 (*N* = 29); the Carrizo Plain population were captured in February 2017 (*N* = 14); and the population in northeastern California were captured in February 2015, February 2016, and January and February 2019 (*N* = 98). This initial set of collars yielded a total of 715,432 points, but these were reduced depending on the modeling approach, described below. Occurrence data were collected from 1999 to 2023 and included observations of pronghorn documented during systematic road and aerial surveys conducted by CDFW staff, harvest locations reported by hunters, and opportunistic observations by CDFW staff, volunteers, and the general public. Given the challenges associated with modeling habitat based on populations that are significantly limited compared to their historical conditions, we adopted an expansive approach with these data sets. We present the combined results to evaluate consensus among them.

We assembled an extensive set of predictor variables, which are believed to either directly influence or act as proxies for pronghorn habitat. These variables were grouped into five categories: roads, soil type, topography, land use/land cover, and freshwater (Table 1, Figure S3). Pronghorn make intricate decisions to maximize their fitness across various spatial and temporal scales (Bean et al., 2024). Typically, they select flat, open terrain in a mix of grass and shrublands (Yoakum, 2004b). In arid regions, pronghorn likely need access to freshwater sources, especially after giving birth and during the driest parts of the year (Bean et al., 2024; Yoakum, 2004c). They are known to exhibit aversion to roads, with their behavior notably altered in proximity to major, heavily trafficked routes (Gavin & Komers, 2006). A recent study additionally highlighted the importance of certain soil characteristics, including bulk density, % clay, and pH, in pronghorn habitat selection (Zeller et al., 2021).

**TABLE 1:** Predictors used for models to estimate pronghorn habitat suitability in California using three techniques: ensemble resource selection functions (“RSF”); step selection functions (“SSF”); and expert opinion (“Expert”).

Predictor	Category	RSF	SSF	Expert	Res.	Aggregation method	Source
Distance to major roads	Roads	Y	Y	Y	10m	Distance	TIGER^a^
Distance to all roads	Roads	N	Y	N	10m	Distance	TIGER
All road density	Roads	Y	Y	N	10m	Density	TIGER
Bulk density	Soil	N	Y	N	30m	Bilinear	Polaris^b^
% clay	Soil	N	Y	N	30m	Bilinear	Polaris
pH	Soil	N	Y	N	30m	Bilinear	Polaris
Elevation	Topography	N	Y	N	30m	Bilinear	NED^c^
Slope	Topography	N	Y	Y	30m	From elevation	NED
TPI	Topography	N	Y	N	30m	From elevation	NED
% agriculture	LULC	Y	Y	Y	30m	% w/in cell	CALFIRE^d^
% barren	LULC	Y	Y	Y	30m	% w/in cell	CALFIRE
% desert	LULC	N	Y	Y	30m	% w/in cell	CALFIRE
% grass	LULC	Y	Y	Y	30m	% w/in cell	CALFIRE
% shrub	LULC	Y	Y	Y	30m	% w/in cell	CALFIRE
% urban	LULC	Y	Y	Y	30m	% w/in cell	CALFIRE

^a^ U.S. Census Bureau (2023).

^b^ Chaney et al. (2019).

^c^ U.S. Geological Survey (2012).

^d^ CalFire (2015).

After preliminary investigation, we opted to exclude all water predictors from our study, despite their known significance to pronghorn survival and reproduction. Our decision was based on difficulty of existing GIS layers to fully capture the extent of freshwater sources accessible to pronghorn to produce a meaningful signal in the model output. Notably, pronghorn heavily rely on and use small free‐standing water sources such as cattle troughs, which are not captured by state‐wide GIS layers (Mohr et al., 2022). This is particularly problematic in arid areas with few documented water sources, as this can lead to non‐sensical results, e.g., that animals are avoiding water when they only use small point sources not recognized in the GIS layer (Mohr et al., 2022). Similarly, fences potentially inhibiting pronghorn movement were not included into the models due to their unavailability at the study area scale. These finer scale drivers of pronghorn habitat suitability can be addressed post modeling effort on a site‐by‐site basis to prioritize acquisition and to conduct reintroductions and habitat management. After assessing these models with the full set of predictor variables (minus the water variables), concerns arose about the possibility of overfitting to the three remaining pronghorn populations. Consequently, we reduced our set of predictors to those we deemed most essential and not previously addressed in the range‐wide models: distance to major roads (federal and state highways and county roads), overall road density, and percent cover of agriculture, barren land, grass, shrub, and urban areas (Bean et al., 2024).

We devised three sets of models of pronghorn habitat, each utilizing different combinations of occurrence data, predictor variables and modeling methodologies. Specifically, we created a set of (1) resource selection functions, (2) step selection functions, and (3) an expert‐driven model (Figure 1, Table 1).

We initially evaluated four sets of resource selection functions using different combinations of occurrences (observations vs. GPS collar data) and predictors (full vs. reduced set). For each combination, we constructed an ensemble model using the *tidysdm* package (Version 0.9.0; Leonardi et al., 2023) in R. This approach facilitated a consistent construction of various modeling techniques into a single consensus model. Specifically, we used Maxent, random forests, generalized linear models with a logistic link, and classification and regression trees. Presences were thinned to a single occurrence per raster cell using the thin_by_cell function in tidysdm, resulting in a total of 14,737 unique occurrences. We used the sample_pseudoabs function to generate background data in a use/availability framework. We generated a number of background points equal to the number of presences, randomly distributed within a 25 km buffer around all presences. We adopted this buffer size as it mirrors the approximate radius of the largest home range reported for arid region pronghorn (Wright & deVos, 1986). All models were executed with default settings in the *tidysdm* package. We present only the results from the GPS collar points using the truncated predictor set. This decision was made because models using observation data, and the model with GPS occurrences and the full set of predictors, appeared to overfit to the existing populations. Results from the other three approaches are available as Supplementary Materials (Figures S4–S13).

Additionally, we employed a step selection function using the *amt* package (Version 0.2.1.0; Signer et al., 2019) in R. For each observed true step, we generated 15 random steps. Pronghorn were filtered to include only those individuals with a minimum of 100 points and uniform sample rates (*N* = 41). Depending on the individual and study area, these rates were set at intervals of 1, 2, 4, or 13 h. Random steps were individually created for each pronghorn. We then compared the actual steps taken (a total of 66,184) to the paired random steps using conditional logistic regression via the clogit function in the *survival* package (Version 3.5.7; Therneau, 2023). Here, we used all predictors (Table 1). It is generally considered inappropriate to extend the findings of a step selection analysis to project landscape‐wide habitat. Decisions made at the individual step level may differ dramatically from those made at the scale of the home range (i.e. comparing 4th order vs. 2nd order, [Johnson, 1980]). Furthermore, the habitat available during a single step is not necessarily indicative of the habitat at coarser scales. Nonetheless, given the inherent challenges of modeling pronghorn in contemporary California, we employed the effect size estimates from the conditional logistic regression to generate a map of suitability.

**FIGURE 2:**
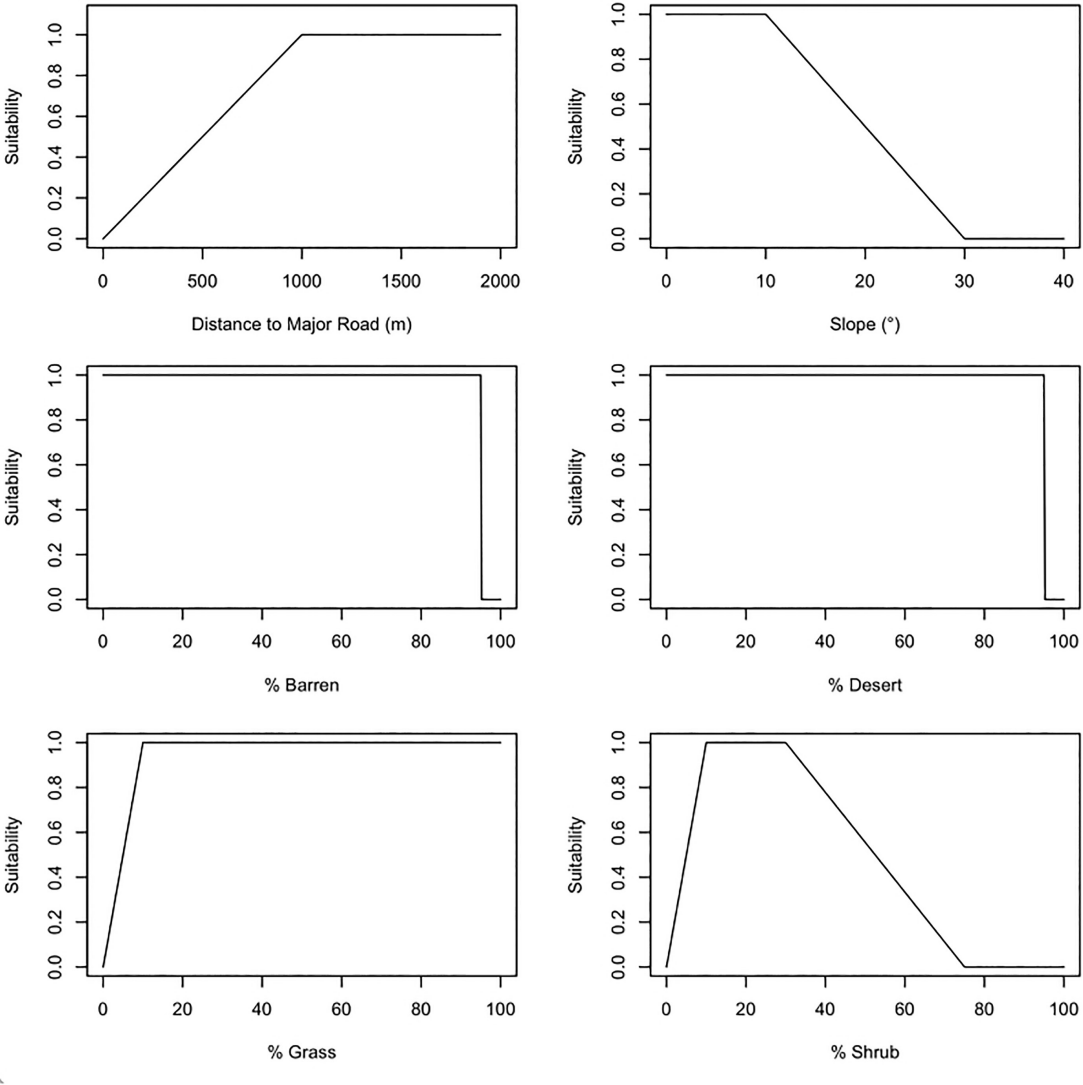
Summary of an expert‐driven model of pronghorn habitat suitability in California. Suitability was defined as a relative value (0–1). The final expert model was calculated for the state by multiplying distance to major road by slope suitability, then multiplying the sum of the 4% land cover categories. Finally, urban and agriculture areas were set to 0.

We subsequently developed an expert‐driven habitat model, grounded in a comprehensive habitat suitability index (Cook & Irwin, 1985; Irwin & Cook, 1985) and a contemporary review of pronghorn literature (Bean et al., 2024). While the original habitat suitability index incorporated several measurements only available in situ (for instance, mean shrub canopy height), we adapted the basic model to accommodate the use of state‐wide GIS data (Figure 2). Our set of predictor variables mirrored the previously mentioned reduced set, with the inclusion of slope. We combined the six set of predictors with the following equation:

$$\left(\text{major roads}\times \text{slope}\right)\times \left(\%\text{barren}+\%\text{desert}+\%\text{grass}+\%\text{shrub}\right).$$

Then, we set all agriculture and urban areas to suitability = 0.

We evaluated each model using either the observation data or GPS collar data, depending on which model was used. Specifically, models formulated with GPS collar data were evaluated against the occurrence data, while the expert model was evaluated using GPS collar data. Performance metrics included the area under the curve (AUC) (Fielding & Bell, 1997) and Boyce Index (Boyce et al., 2002; Hirzel et al., 2006). We present these metrics, recognizing them as standard benchmarks in habitat modeling. However, it is crucial to highlight the challenges inherent in evaluating these models given the significant contraction of pronghorn range in California. Models of species occupying a small portion of their potential niche can appear more accurate than they actually are (Bean et al., 2012). It is plausible that vast stretches of land historically inhabited by pronghorn could become viable again with concerted restoration efforts (e.g., adding free‐standing year‐long water, modifying fences to allow pronghorn movement, shrub restoration for fawn protection and more dependable year‐round forage).

### 2.3 Final consensus

We combined these habitat models into a unified set of consensus maps. We converted each continuous model of habitat suitability into a set of binary habitat/non‐habitat classes. Then, we clipped the models of habitat to the potential contemporary and potential future climate range. We added the binary maps together to highlight areas where 1, 2, or all 3 models found habitat. Additionally, to guide restoration and reintroduction efforts, we superimposed current urban and agricultural areas, regions of conflicting land use relative to pronghorn conservation, to eliminate from consideration in any land acquisition or reintroduction planning.

## 3 RESULTS

### 3.1 Potential climate distribution

We developed models based on two distinct hypotheses: one positing that pronghorn in California historically migrated to escape extreme climate conditions (Supplementary Materials ODMAP SDM Migration), and the other suggesting that pronghorn experienced a niche reduction (ODMAP Niche Reduction).

**FIGURE 3:**
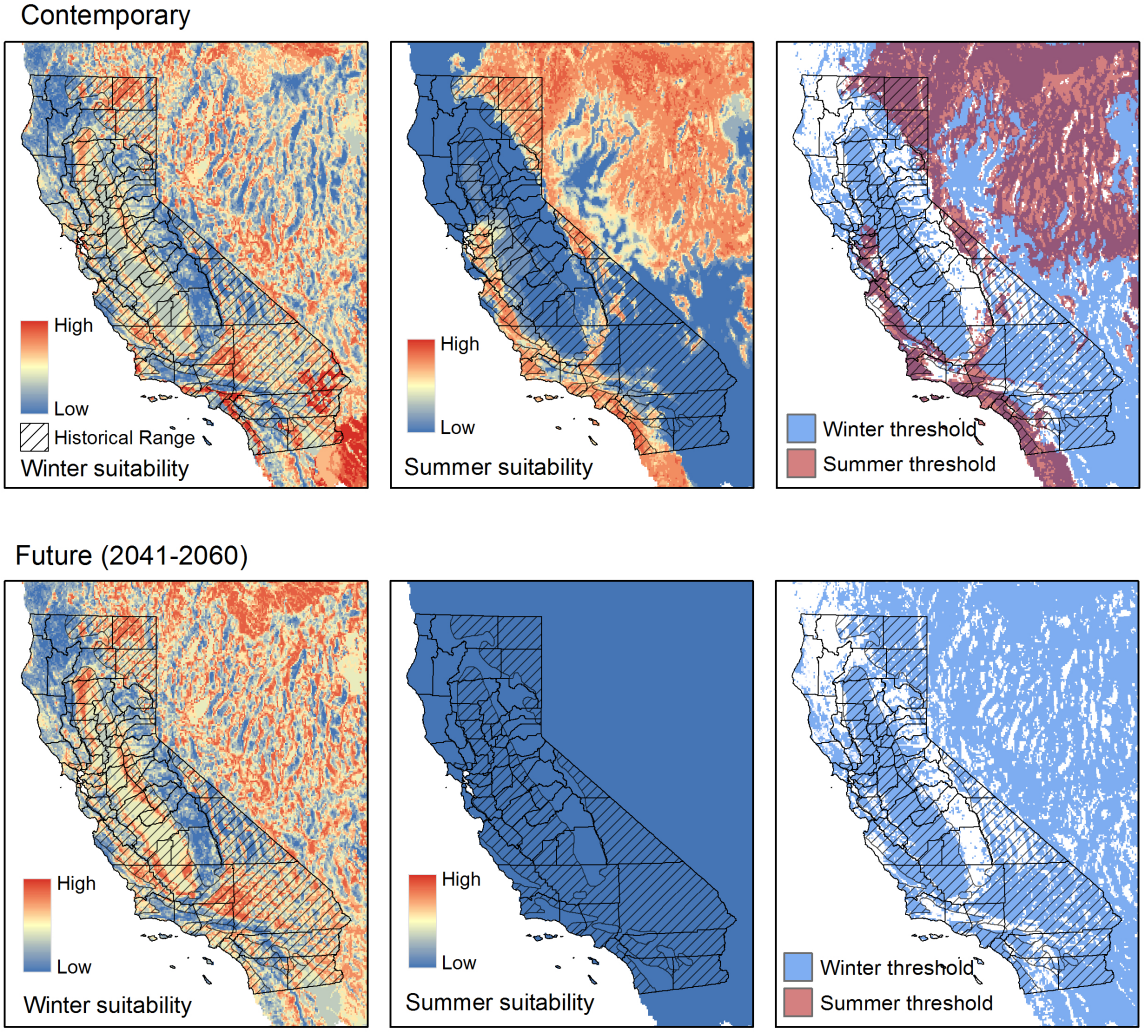
(Top) Estimated contemporary climate suitability for pronghorn under the assumption that individuals in California migrated seasonally to escape extreme cold (left) and/or extreme cold (middle); (right) winter and summer models where values were ≥15th percentile of all values where pronghorn were found in that season. (Bottom) Contemporary models projected onto 25 future global circulation models, mean for winter (left) and summer (middle); (right) future winter and summer models where values were ≥15th percentile of all values where pronghorn were currently found in that season.

According to the migratory hypothesis, we found that pronghorn in California might have been historically restricted from inhabiting the San Joaquin Valley of southern California in summer due to extreme high temperatures (Figure 3). However, during winter, most of the state's flatter regions would have been suitable (Figure 3). Across their entire range, pronghorn seem limited to areas with winter mean minimum temperatures > −10°C and terrains with slopes <23°. During the summer, pronghorn were associated with areas with annual precipitation <100 cm, slopes <11.5°, and mean maximum temperatures ranging between 10 and 36°C (Figures S1 and S2). If this hypothesis holds true, pronghorn would have historically migrated from the inner coast ranges or Sierra foothills during the summer to the San Joaquin Valley of southern California in winter. Under this hypothesis, by 2041–2060, while winters are expected to remain hospitable, summer temperatures might exceed sustainable thresholds for pronghorn (Figure 3). However, while winter climate projections were highly similar to contemporary temperatures, the summer projections were entirely dissimilar (Figure S17).

**FIGURE 4:**
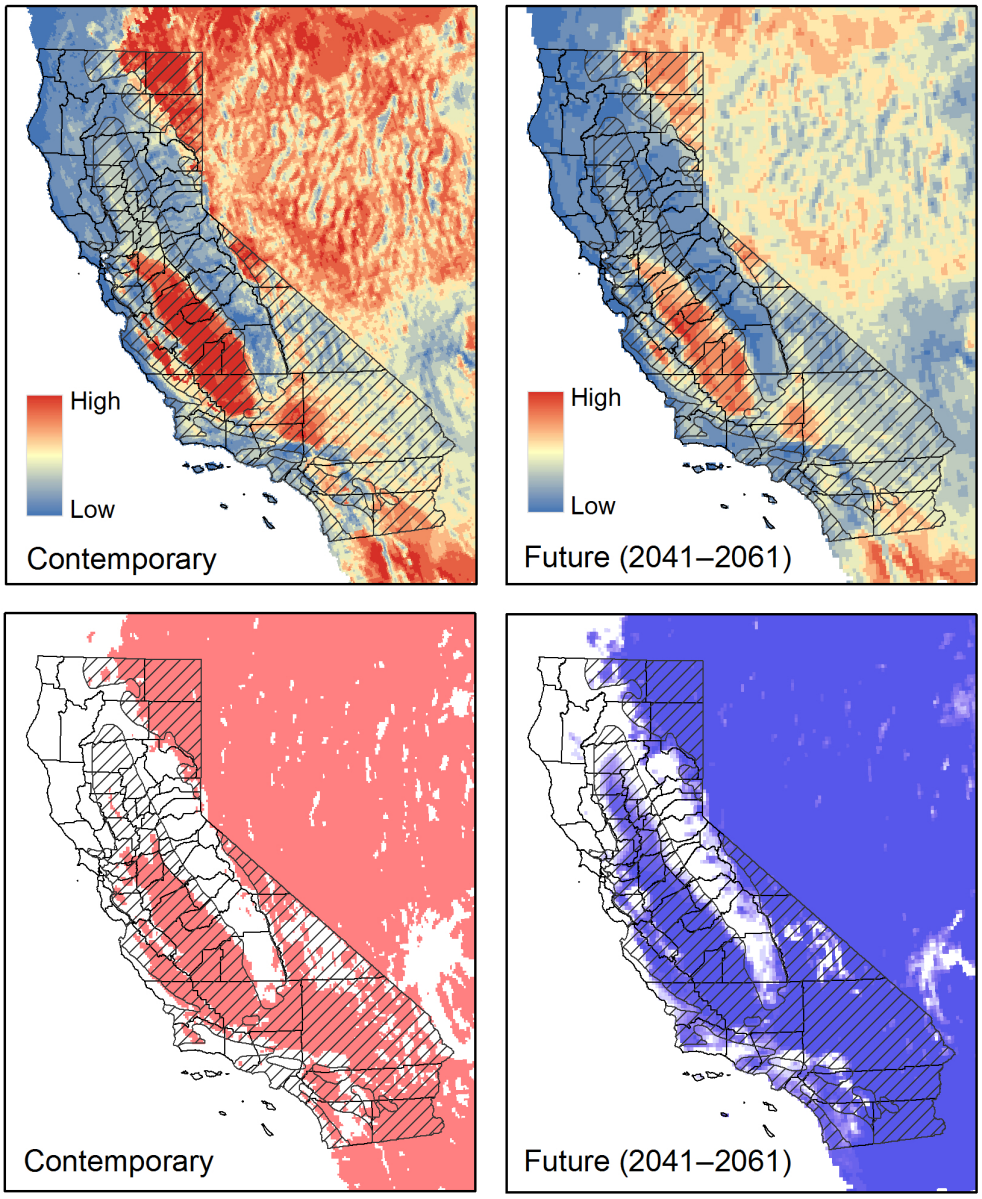
Estimated climate suitability under the assumption that pronghorn in California have undergone an environmental niche reduction. Contemporary model (left) estimated using all pronghorn occurrences from the Global Biodiversity Information Facility pre‐1970 and an additional 20 points randomly selected from contemporary Sonoran pronghorn occurrences. Future estimate (top right) shows mean suitability from projection of contemporary model onto 25 global circulation models. Bottom left shows all areas of climate suitability greater than the value found at 95% of all pronghorn occurrences; bottom right shows the summation of that threshold value for each of the 25 global circulation models (i.e. lighter blues = fewer global circulation models met the threshold at that location; darker blues = more models met the threshold).

Under the niche reduction hypothesis, a larger portion of California emerged as having a suitable climate, both now and into the future, including the Sacramento Valley of northern California and a new focal area for acquisition and management by The Nature Conservancy, California Department of Fish and Wildlife, and partners, the San Andreas Linkage (S. Butterfield, personal communication, December 11, 2023) located north of the Carrizo Plain and extending through Henry Coe State Park near San Jose, California (2041–2060; Figure 4). The extreme ends of the Great Central Valley appeared to have novel climate conditions, particularly the southern and eastern ends of the San Joaquin Valley and the northern end of the Sacramento Valley (Figure S17), as did the southeastern part of the state.

### 3.2 Habitat suitability

**FIGURE 5:**
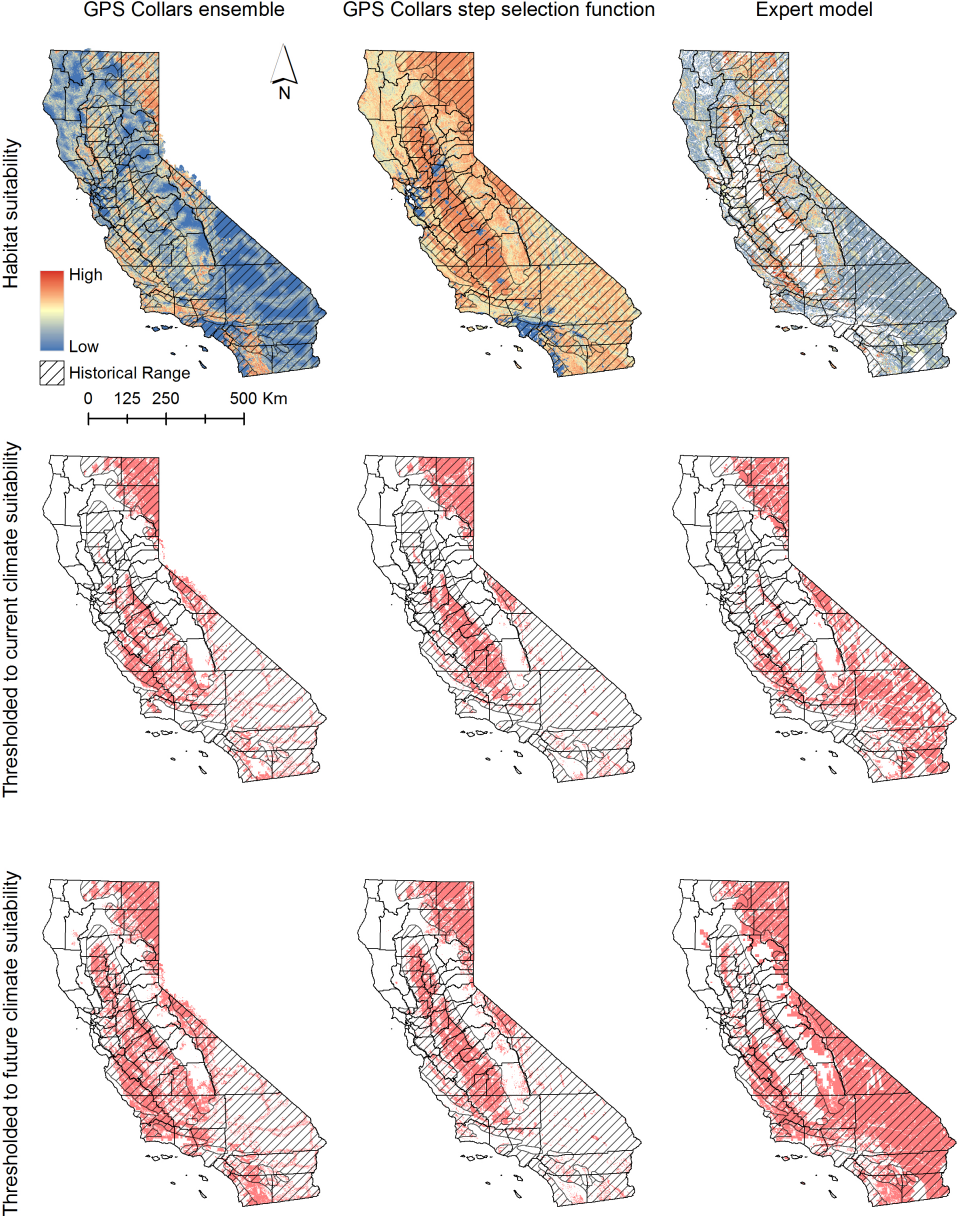
Three approaches to modeling contemporary pronghorn habitat suitability in California. (Left) Mean habitat suitability modeled using GPS telemetry data from three populations (Modoc, Bodie Hills, and Carrizo) and an ensemble of Maxent, random forests, classification and regression trees, and a generalized linear model with a logistic link; (middle) Step selection function using GPS telemetry data from three populations; (right) an expert‐driven model. Middle row is the habitat suitability models thresholded to the top 99% of all pronghorn occurrences and then further clipped to the contemporary climate suitability estimated from a historical model. Bottom row shows the habitat suitability models thresholded to the top 99% of all pronghorn occurrences and then clipped to the future climate suitability.

We employed three different strategies to model contemporary pronghorn habitat in California: first, by integrating pooled GPS telemetry data with an ensemble of Maxent, random forests, boosted regression trees, and a generalized linear model with a logistic link; secondly, by utilizing GPS telemetry data from individual pronghorn combined with a step selection function; and thirdly, through an expert‐driven model (Figure 5).

According to both the ensemble and step selection models, habitat for pronghorn currently exists in northeastern California, within Mono and Inyo Counties, and across the San Joaquin Valley of southern California (Figure 5). When employing pooled GPS telemetry data with Maxent, pronghorn appeared to select areas with a higher percentage of grassland, shrubland, and barren terrains. They also seemed to select areas with lower urban coverage and closer to major roads (Figure S14). The GPS ensemble model yielded a Boyce Index of 0.99 and an AUC score of 0.87.

According to the output of the step selection function, pronghorn selected areas closer to roads but in areas of lower road density. They selected ridgetops, as indicated by higher values of the topographic position index, and areas of lesser terrain ruggedness. They selected greater cover of agriculture, barren, grassland and shrubland, and lower cover of desert and urban (Figure S15). The GPS step selection function yielded a Boyce Index of 0.74 and an AUC score of 0.93.

Distinctly, the expert model diverged from the other 2 habitat models in two aspects. Primarily, this approach identified substantially more habitat in the Mojave Desert. Conversely, it indicated much less habitat in the agriculturally dominated San Joaquin Valley of southern California. The Boyce Index for the expert model was 0.87 and the AUC was 0.63.

### 3.3 Final consensus

**FIGURE 6:**
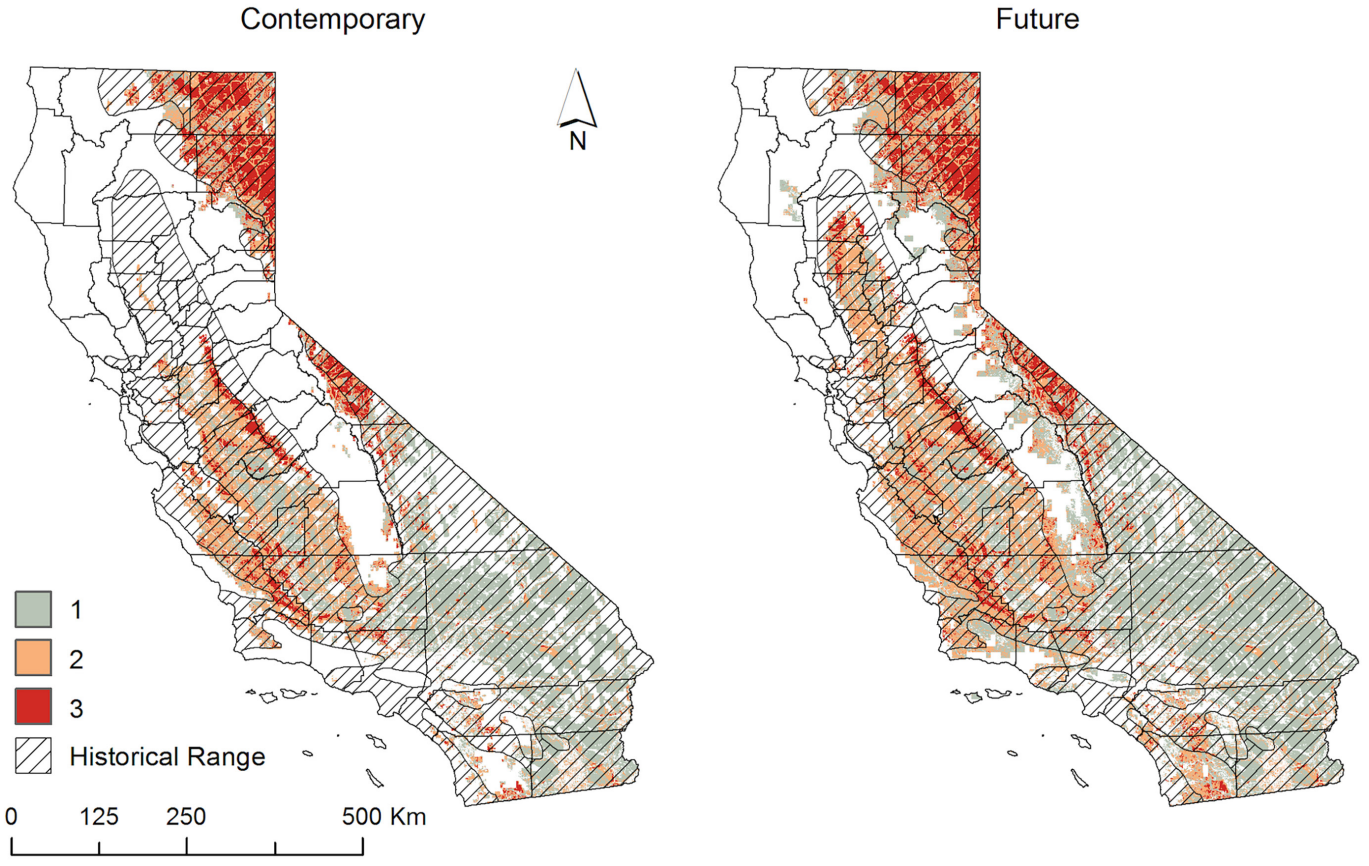
Consensus among three models of pronghorn habitat in California (ensemble model using four statistical approaches; step selection function; and an expert‐driven model), clipped to contemporary and future climate suitability. Values represent the number of habitat models (0, 1, 2 or 3) predicted that area as suitable. White areas represent either areas where climate is not predicted suitable, habitat was not predicted present, or both.

We identified spatial congruency across models to describe areas of consensus among the three modeling approaches (Figure 6 and Figure S16). Areas of contemporary consensus among all three models included northeastern California, Mono and Inyo Counties, and the peripheries of the San Joaquin Valley of southern California that have not been converted to agriculture. Furthermore, there was a band of currently unoccupied habitat, approximately 200 km in length, situated in the central western Sierra Foothills. In the future, the northern perimeter of the Sacramento Valley of northern California is projected to emerge as climatically suitable. Slightly more than 18% of California was predicted as suitable habitat by 2 (52,324 km^2^) or 3 (25,860 km^2^) out of the 3 models (though varying combinations of model consensus were observed in different regions). This included the Salinas Valley and surrounding areas, the Santa Ynez Valley in Santa Barbara County, and portions of Kern County both to the north and south of the Tehachapi Range. Additionally, there were small patches of isolated habitat in eastern San Diego and western Riverside counties.

## 4 DISCUSSION

Identifying areas suitable for pronghorn habitat acquisition, reintroduction, and management is inherently difficult because some foundational assumptions of suitability or distribution models are a priori violated. We therefore attempted to understand current and future potential suitability for pronghorn in California using a multiscale, multistep model. Under one scenario (the niche reduction hypothesis), climate in California can continue to support pronghorn now and into the future—in fact, areas of suitable climate may even expand beyond what was historically occupied prior to European colonization. Under the other (migration), summers will become too hot for pronghorn anywhere in the state. At a finer scale, suitability models using either observation or GPS collar data initially appeared to overfit to the remaining populations. We overcame this obstacle in three different ways: by using a reduced set of predictors; using a finer‐scale estimate of habitat selection (step selection function); and relying on a traditional technique for identifying suitable habitat through expert opinion. These three models produced different estimates of pronghorn habitat in California, but the consensus among them helped to identify a few large landscapes for managers to focus on.

The outcomes of the climate models have serious implications for pronghorn conservation in California in the face of climate change. If the San Joaquin Valley of southern California were historically too hot in summer, forcing pronghorn to migrate into cooler and wetter climates, it appears that climate change will make California inhospitable for the species. Unfortunately, we know of no historical evidence for migration (but it is intriguing to note that the combined winter and summer models appear to overlap well with the estimated historical range). Reviving pronghorn migration to southern California would likely be a much greater undertaking than reintroduction, assuming of course that stable or growing source populations of pronghorn genetically fit for hotter and drier habitats are available. There are uncountable barriers to long‐distance migration throughout the state in the form of major roads and fences, although presently The Nature Conservancy, California Department of Fish and Wildlife, and partners are focused on a long‐term acquisition and management strategy that would begin to connect southern California pronghorn to central and northern potentially suitable habitat (S. Butterfield, personal communication, December 11, 2023). Further, ungulates appear to lose the knowledge required to migrate after extirpation and reintroduction and may take decades or longer to evolve again (Jesmer et al., 2018). Future work into the historical ecology of the species in California would help to resolve whether migration was a part of pronghorn life history (e.g., Jefferson & Goldin, 1989).

In addition, a mechanistic species distribution model for pronghorn could more directly address their physiological limitations imposed by the hot and dry conditions in California (Buckley et al., 2010; Evans et al., 2015). While these models have been used less frequently with endotherms (Kearney & Porter, 2009), most inputs required to construct a model are available for pronghorn and would provide needed, spatially explicit information about their energetic needs. This could also address the challenges of projecting into novel climate spaces. In the contemporary model, pronghorn suitability dropped to 0 above approximately 40°C mean daily maximum. Projecting into future scenarios, we assumed that suitability would continue to be low above those temperatures, however our model of summer climate distribution under the migratory hypothesis was projected into entirely dissimilar climate space. A model based in the physiology of the animal, and the microclimates available (i.e., shade) would better address these limitations.

In the meantime, the niche reduction climate model also closely matched the historical distribution of the species, and evidence for a reduction in niche space is better supported in the literature (Bean et al., 2024; Brown et al., 2006). Both model outputs, and other analyses of the species' climatic niche (Bean et al., 2024) suggest that any future assisted reintroduction of pronghorn in California should source from other populations occupying more similar climatic conditions, such as in New Mexico or Texas or even from more arid‐adapted subspecies like Sonoran or Peninsular pronghorn to better match the current and future climate regime. A better understanding of the genetic stock within populations across the arid Southwest is needed to inform future reintroductions (Klimova et al., 2014; Lee et al., 1989; Stephen et al., 2005).

There were two additional concerns with the climate data used in our models. There was some uncertainty about suitable climate in the future in the southern and eastern San Joaquin Valley and northern Sacramento Valley, as well as the eastern Mojave Desert. In those areas, future climate was highly dissimilar from nearby contemporary climate space, making it more difficult to project pronghorn response (Elith et al., 2010). Additionally, there was a slight mismatch in the temporal extent of our pronghorn occurrence data and climate. Climate data for pronghorn range was only available as 30‐year averages from 1970 to 2000, but we had very few occurrences available from the 1970s. Pronghorn records from after 2000 therefore may have appeared in the models to occupy environments cooler and wetter than they were experiencing. In that case, our projections would likely underestimate future suitable climate.

Our habitat models differed from previous attempts to identify pronghorn habitat in other parts of their range. In particular, we found in both models using GPS data (the resource selection function and step selection function), pronghorn appeared to select areas closer to major roads, similar to findings by Leu et al. (2011), but in contrast to previous models that found pronghorn avoiding areas with high density of roads (Burroughs, 2013) or selecting areas farther away from roads (Christie et al., 2017; Duncan et al., 2016; Poor et al., 2012). In one other study, pronghorn avoided roads in winter but selected them in summer (Reinking et al., 2019). It may be that major roads are confounded with flatter terrain in areas where pronghorn remain, or the roads in these remote regions of the state have less traffic.

Similarly, many of our models (including those not presented in the main text) found pronghorn selected areas with higher percent cover of agriculture. This is a result consistent with some studies of pronghorn habitat selection (Selting & Irby, 1997), but not others (Christie et al., 2017; Duncan et al., 2016; Jakes et al., 2020; Zeller et al., 2021). We note that pronghorn have a complicated relationship with agriculture. While pronghorn are known to occasionally utilize cultivated crops (e.g., alfalfa) to meet seasonal nutritional requirements, use is generally periodic (i.e., focused during hotter and drier times of the year when natural vegetation forage sources are more limited in more arid populations or during the winter when snowpack prevents access to preferred forage) and in juxtaposition to more natural cover types (e.g. lands entered into the Conservation Reserve Program) or suitable rangeland habitat (Opatz, 2020; Yoakum, 2004b). In southern California, pronghorn use these fallowed dry farmed areas in the summer when food sources are more limiting and non‐native “weedy” forbs present a viable food source. They may also be selecting areas with more water availability. The Colusa‐Glenn reintroduced population northwest of Sacramento in northern California may present an extreme case of reliance on agriculture to meet nutritional needs year‐round. This pronghorn population, believed to consist of no greater than 30–40 individuals, almost exclusively occupies cultivated cropland, notably using fruit and nut tree orchards (substantiated by photographs; CDFW internal data). This population may therefore be nutritionally supplemented by agricultural forage or “weedy” species associated with agricultural edge habitat (i.e., rice levees, orchard undergrowth) in the presence of deficient surrounding rangeland habitat. The species' complicated relationship with certain resources on agricultural settings suggest that “agricultural” as a monolithic land cover type may not adequately explain space use patterns. The use of agricultural food resources during hotter and drier summer months does highlight that any acquisition/reintroduction/management strategy for pronghorn must also focus on habitat restoration for year‐long forage, including possibly shrub restoration, which could additionally allow for cover for fawns during sensitive time periods when fawns are small and move more slowly and thus are more susceptible to predation.

Our models did not incorporate two other major barriers to pronghorn success: fences and free water availability. Although recent advances suggest a way forward for identifying fences at a landscape scale (Poor et al., 2014), we did not have a state‐wide estimate of fences that could block pronghorn movement. Fences likely preclude the Sierra foothills as a place for reintroduction, despite the predicted habitat available in the region. At a finer scale, fence removal and modification is a viable strategy for supporting pronghorn populations (Yoakum, 2004b). Similarly, lack of data on small freshwater sources prevents their inclusion in the habitat models. Acquisition/reintroduction/management efforts will similarly need to identify available water and ensure they are available at recommended scales (Yoakum, 2004b).

The use of correlative models in ecology and conservation has exploded over the past two decades. However, their use to prioritize areas for acquisition, reintroduction, and habitat management is complicated by a number of factors. This is particularly true when habitat selection is studied in previously reintroduced populations. Reintroduced populations may have mismatched genotypes; incomplete social knowledge about resource timing and availability; and they often occupy environments altered from historical norms (Frankham et al., 2017). Inferring habitat selection that can maximize fitness from these populations may give incomplete or even incorrect information about their habitat needs.

In this study, we used a multiscale, multistep approach to modeling habitat. In doing so, we identified new questions required to understand the conservation of pronghorn into the next century—did they migrate in southern California as they do in northeastern parts of the state? If so, was migration driven by temperature or resources (so that optimal habitat management might mitigate the need for restoring migration)? Are there existing populations that can tolerate the projected extreme heat and aridity in the state? We used a multifaceted model of habitat to identify areas of consensus that, taken together, will help better identify areas for managers and conservation scientists to support acquisition, natural and assisted recolonization, and management of habitat for this iconic North American species. While these models and their outputs can be used as a guide towards pronghorn conservation, ultimately on the ground efforts will need to take the next step in ensuring pronghorn stability and hopefully recovery across their California range in the future.

## AUTHOR CONTRIBUTIONS

**William T. Bean:** Conceptualization (equal); data curation (equal); formal analysis (lead); funding acquisition (supporting); investigation (lead); methodology (equal); project administration (equal); resources (equal); validation (equal); visualization (equal); writing – original draft (lead); writing – review and editing (equal). **H. Scott Butterfield:** Conceptualization (equal); funding acquisition (equal); investigation (equal); methodology (equal); project administration (equal); resources (equal); supervision (equal); writing – review and editing (lead). **Jeanette K. Howard:** Conceptualization (equal); funding acquisition (equal); investigation (equal); methodology (equal); project administration (equal); resources (equal); supervision (equal); writing – review and editing (equal). **Thomas J. Batter:** Conceptualization (equal); data curation (lead); investigation (equal); methodology (equal); project administration (equal); writing – review and editing (equal).

## FUNDING INFORMATION

Funding for this work was through a grant from The Nature Conservancy and in kind support from Cal Poly, The Nature Conservancy, and California Department of Fish & Wildlife.

## CONFLICT OF INTEREST STATEMENT

The authors declare that there are no conflicts of interest.

## Supporting information


Data S1.

## Data Availability

The Data that support the findings of this study are openly available in Dryad at: https://doi.org/10.5061/dryad.bcc2fqzmx.
