# Supplementary material for: Climatically robust multiscale species distribution models to support pronghorn recovery in California
Source: Ecol Evol. 2024 Jun 20;14(6):e11454. doi: 10.1002/ece3.11454 (PMC11188984; doi:10.1002/ece3.11454)
Supplement: Supplementary file 1 — Data S1. [file ECE3-14-e11454-s001.zip › ece311454-sup-0001-Supplementary Figure S1-S17.docx]

**Pronghorn Supplementary Material**

**Data availability link (for review only):** <https://drive.google.com/drive/folders/1qfIt8fy0gdT7xl5Wea4ypKKOI3fGBIXb?usp=sharing>

**Species Distribution Model – Migration Hypothesis**


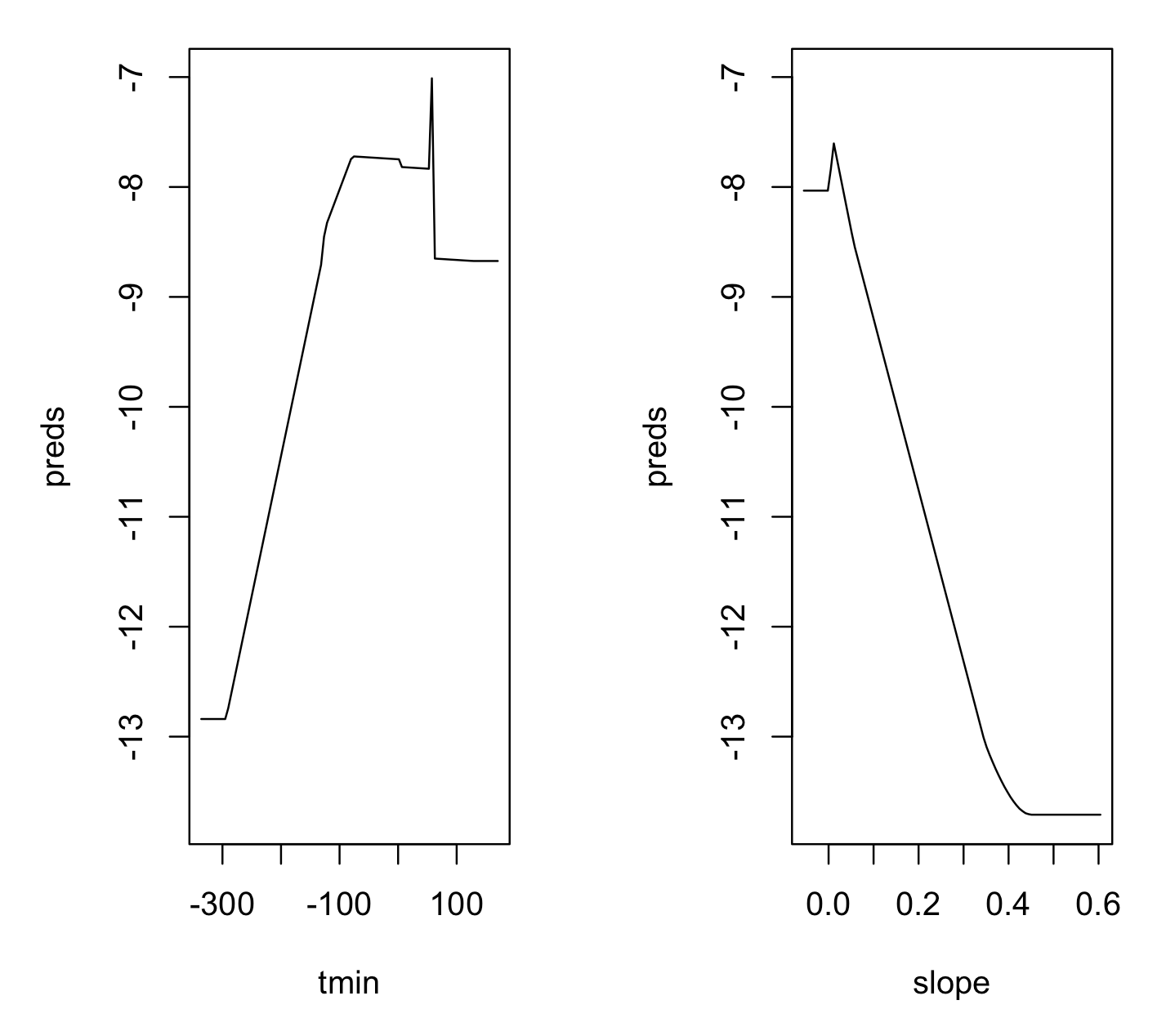


Figure 1. Response curves from a model of winter pronghorn distribution from all occurrences collected >=1970, November-February; tmin = mean minimum temperature (in °C*10) for the month in which the occurrence was recorded; slope in radians.


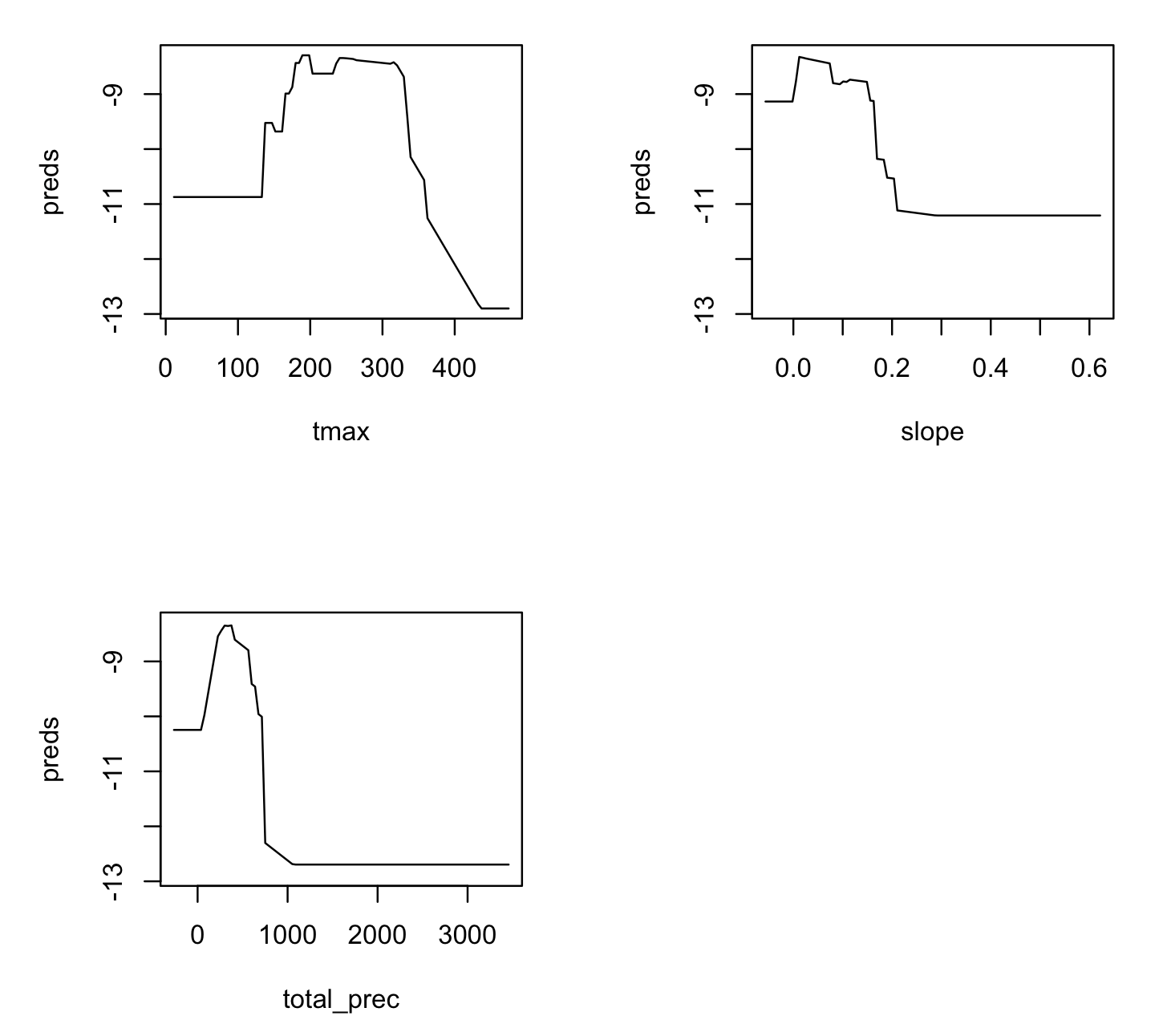


Figure 2. Response curves from a model of summer pronghorn distribution from all occurrences collected >=1970, May-September; tmax = mean maximum temperature (in °C*10) for the month in which the occurrence was recorded. Total precipitation (total_prec) in mm, slope in radians

**Habitat Suitability Models**

**
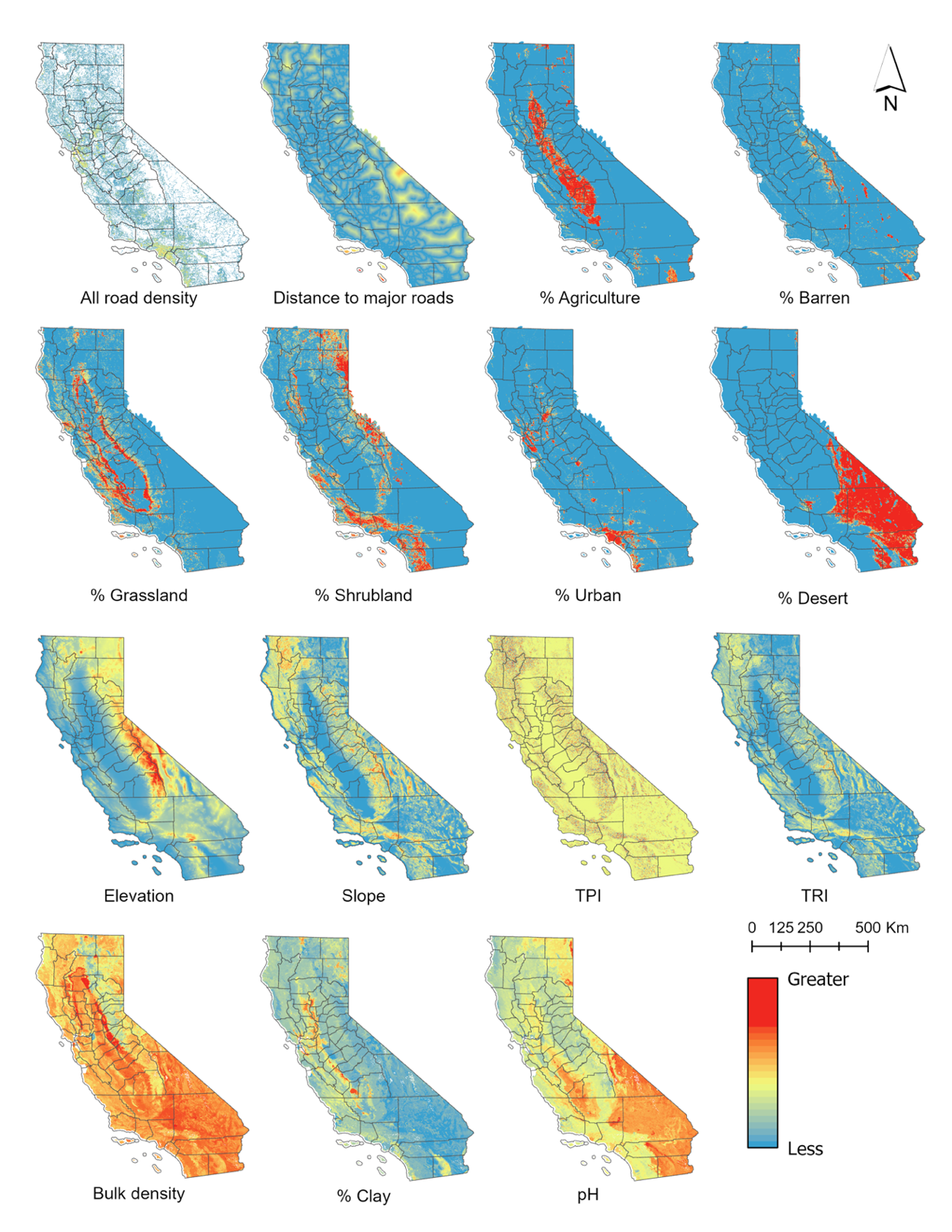
**

Figure 3. Environmental predictors used to estimate pronghorn habitat in California.


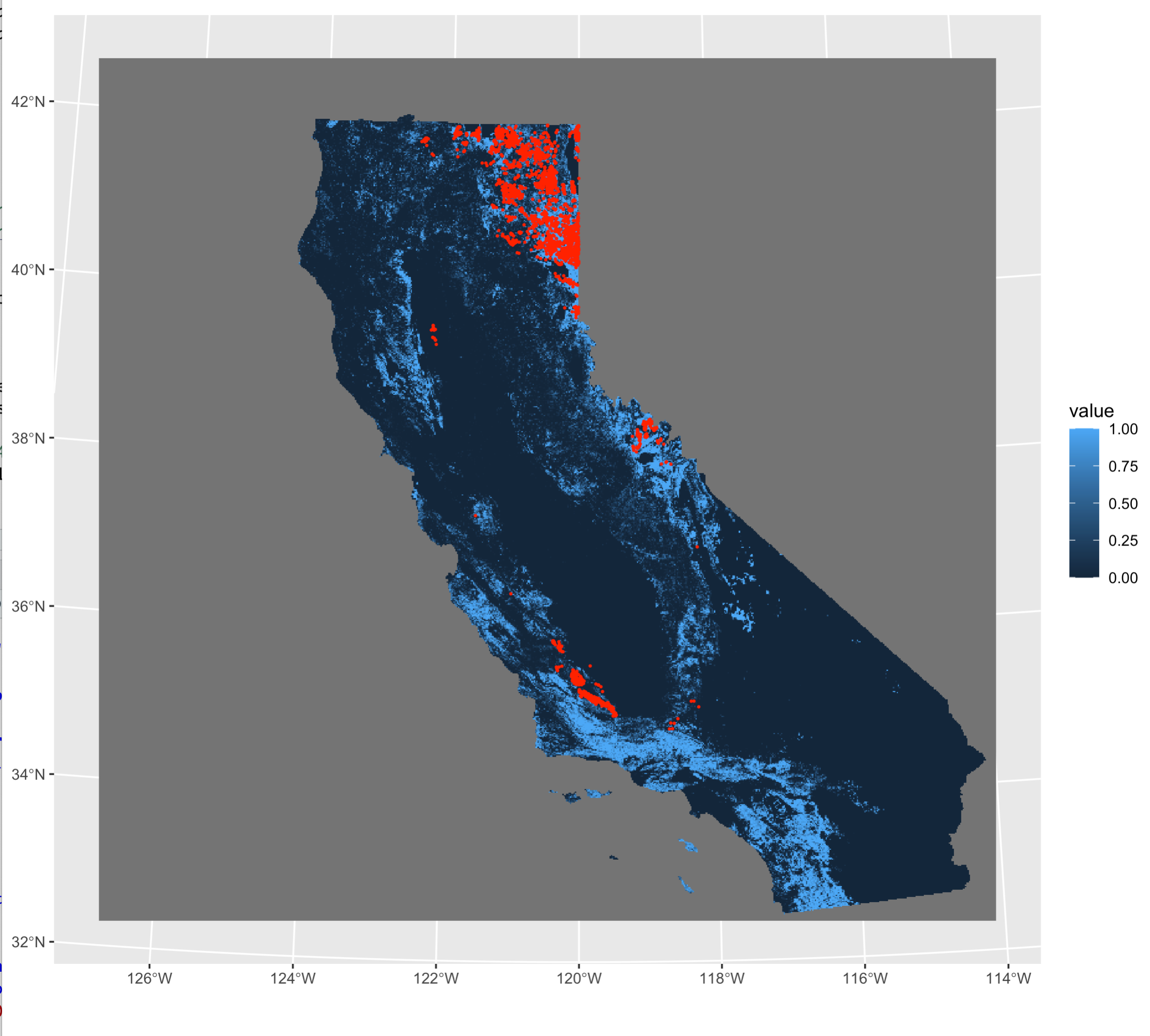


Figure 4. Pronghorn observations from CDFW in red; proportion of shrub cover in blue (calculated as the proportion of 30 m x 30 m raster cells classified as “shrub” in the CalFire FRAP vegetation dataset within a 540 m x 540 m area).


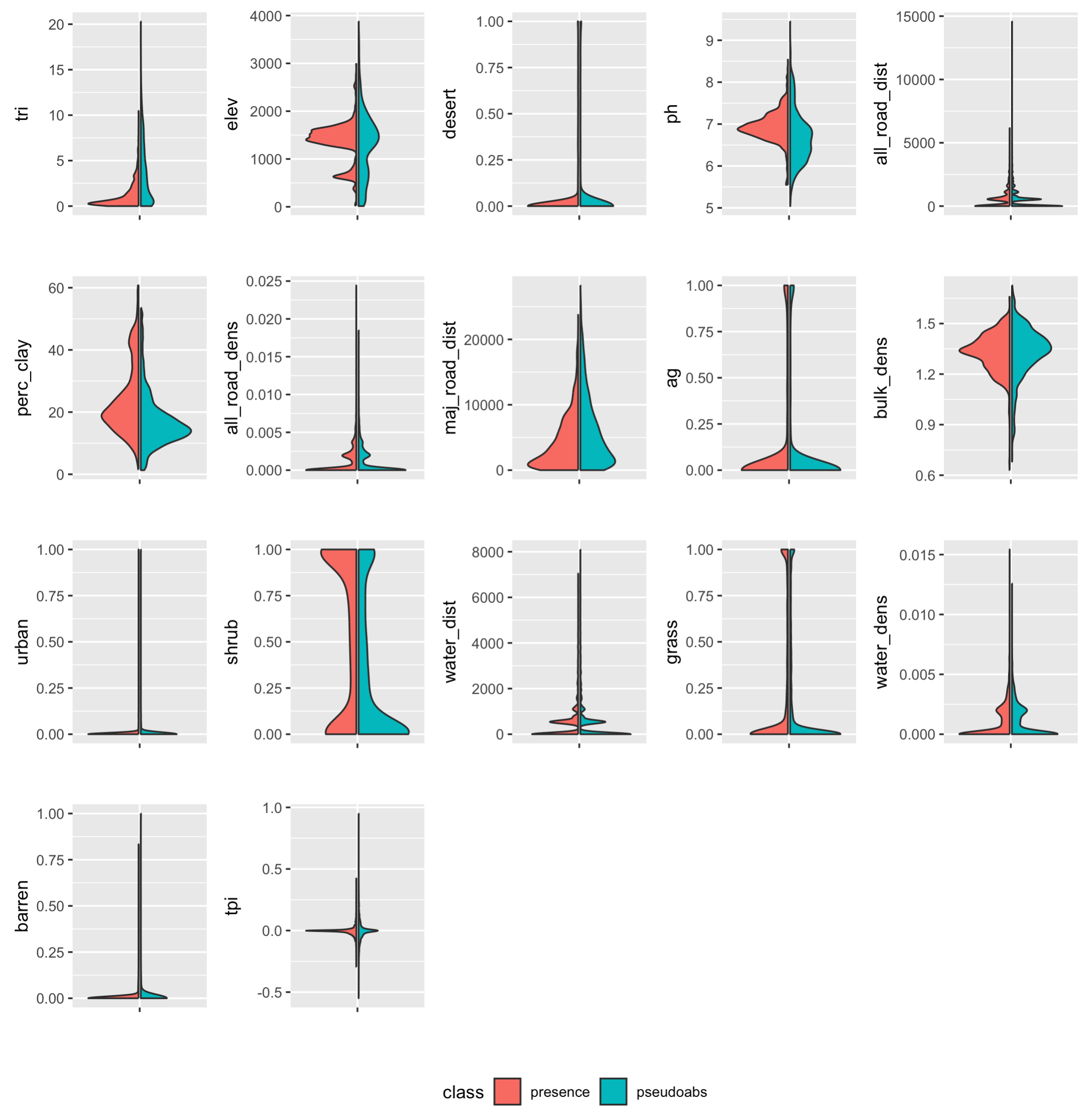


Figure 5. Distribution of predictor values at pronghorn observations collected by CDFW (red) and background points randomly generated within 25 km of occurrences. Tri=terrain ruggednes index; elev = elevation; desert = proportion of cells classified as desert; ph = pH of soil; all_road_dist=distance to any road in TIGER lines database; perc_clay = percentage clay in soil; all_road_dens = density of all roads; maj_road_dist = distance to major roads (federal and state highways and county roads); ag = proportion of cells classified as agriculture; bulk_dens = bulk density of soil; urban = proportion of cells classified as urban; shrub = proportion of cells classified as shrub; water_dist = distance to freshwater; grass = proportion of cells classified as grassland; water_dens = density of freshwater cover within cell; barren = proportion of cells classified as barren; tpi = topographic position index


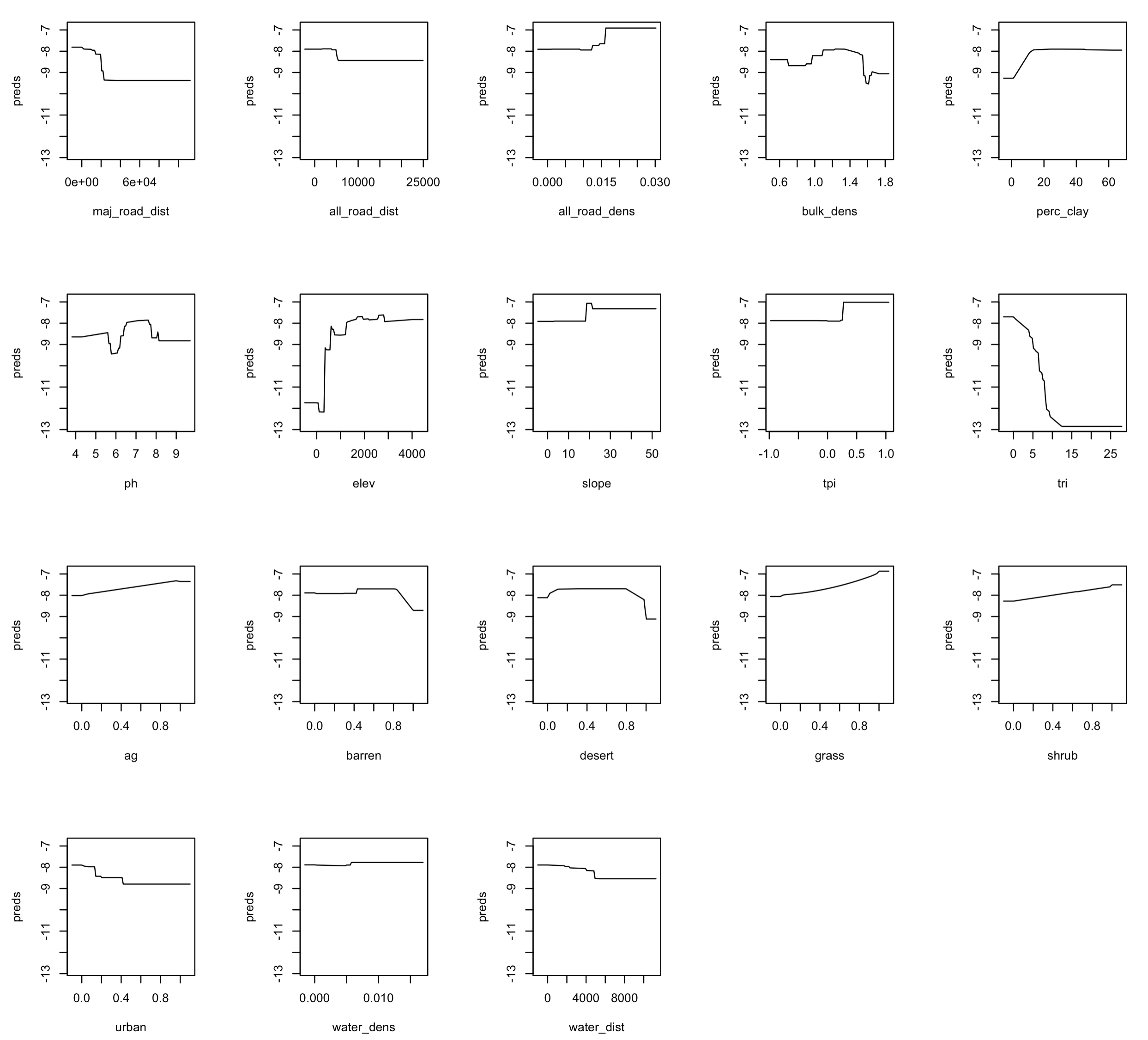


Figure 6. Response curves from Maxent-based habitat suitability model for pronghorn using occurrences collected by CDFW and full set of predictors. Maj_road_dist = distance to major roads (federal and state highways and county roads); all_road_dist=distance to any road in TIGER lines database; all_road_dens = density of all roads; bulk_dens = bulk density of soil; elev = elevation; perc_clay = percentage clay in soil; ph = pH of soil; slope = slope in degrees; tpi = topographic position index; tri=terrain ruggednes index; ag = proportion of cells classified as agriculture; barren = proportion of cells classified as barren; desert = proportion of cells classified as desert; grass = proportion of cells classified as grassland; shrub = proportion of cells classified as shrub; urban = proportion of cells classified as urban; water_dens = density of freshwater cover within cell; water_dist = distance to freshwater.


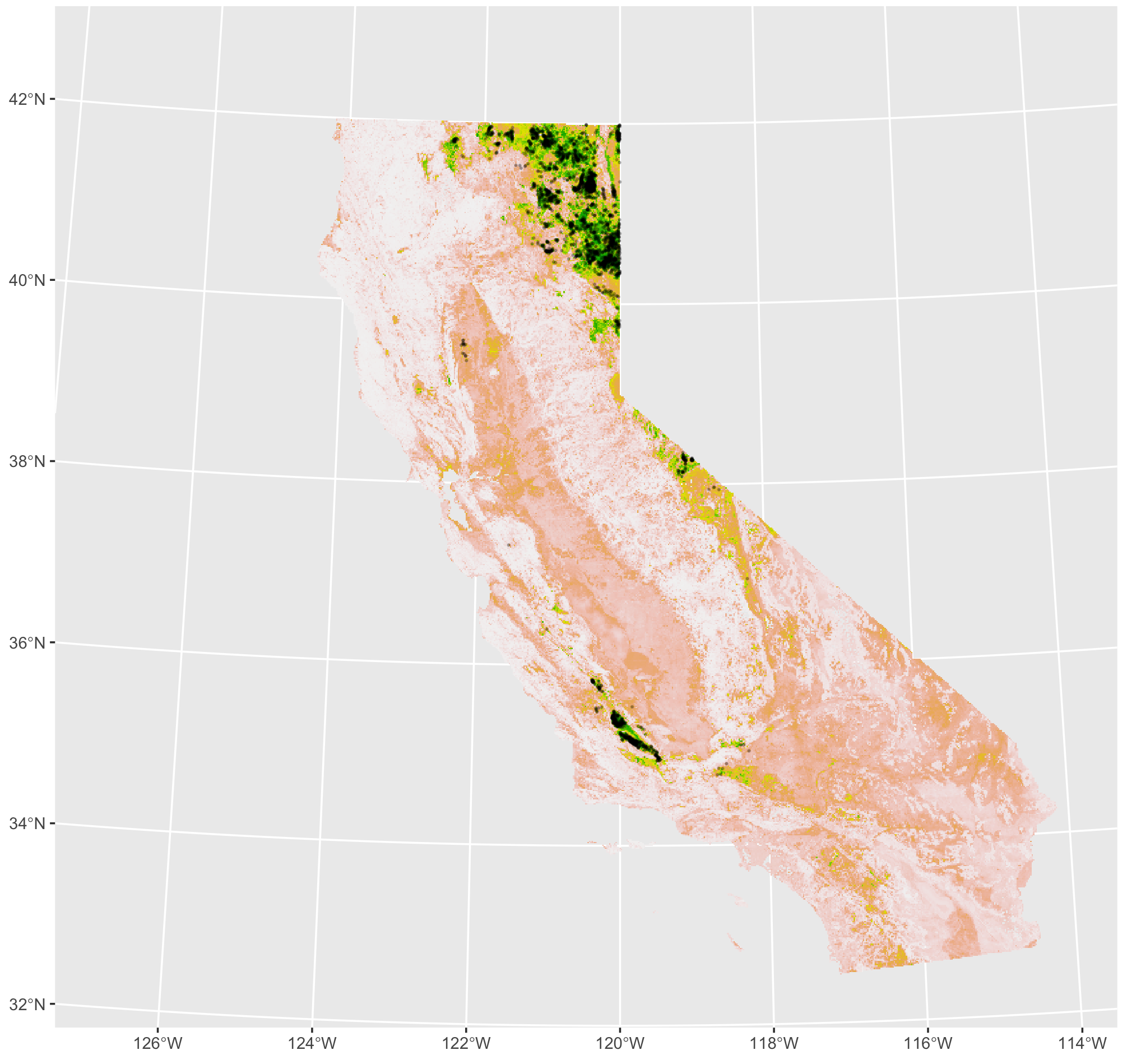


Figure 7. Predicted pronghorn habitat from ensemble of habitat suitability using Maxent, Random Forests, Classification and Regression Trees, and a Generalized Linear Model with a logistic link. Green = high suitability, white = low suitability; black points are observations collected opportunistically by CDFW used in model.


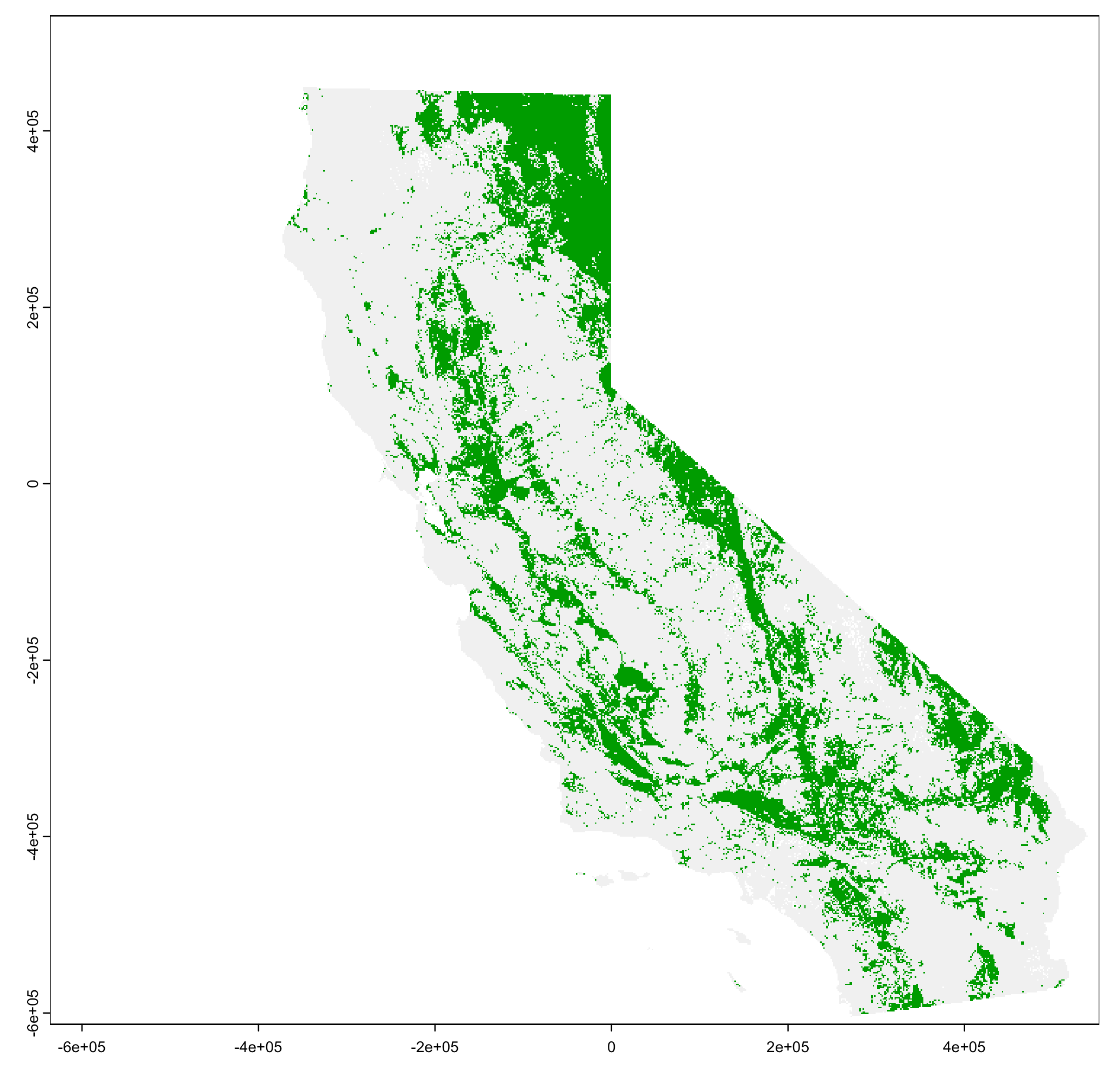


Figure 8. Ensemble model of pronghorn habitat suitability using occurrences collected opportunistically by CDFW; green areas represent all areas with suitability value >= any pronghorn occurrence in database used to train model.


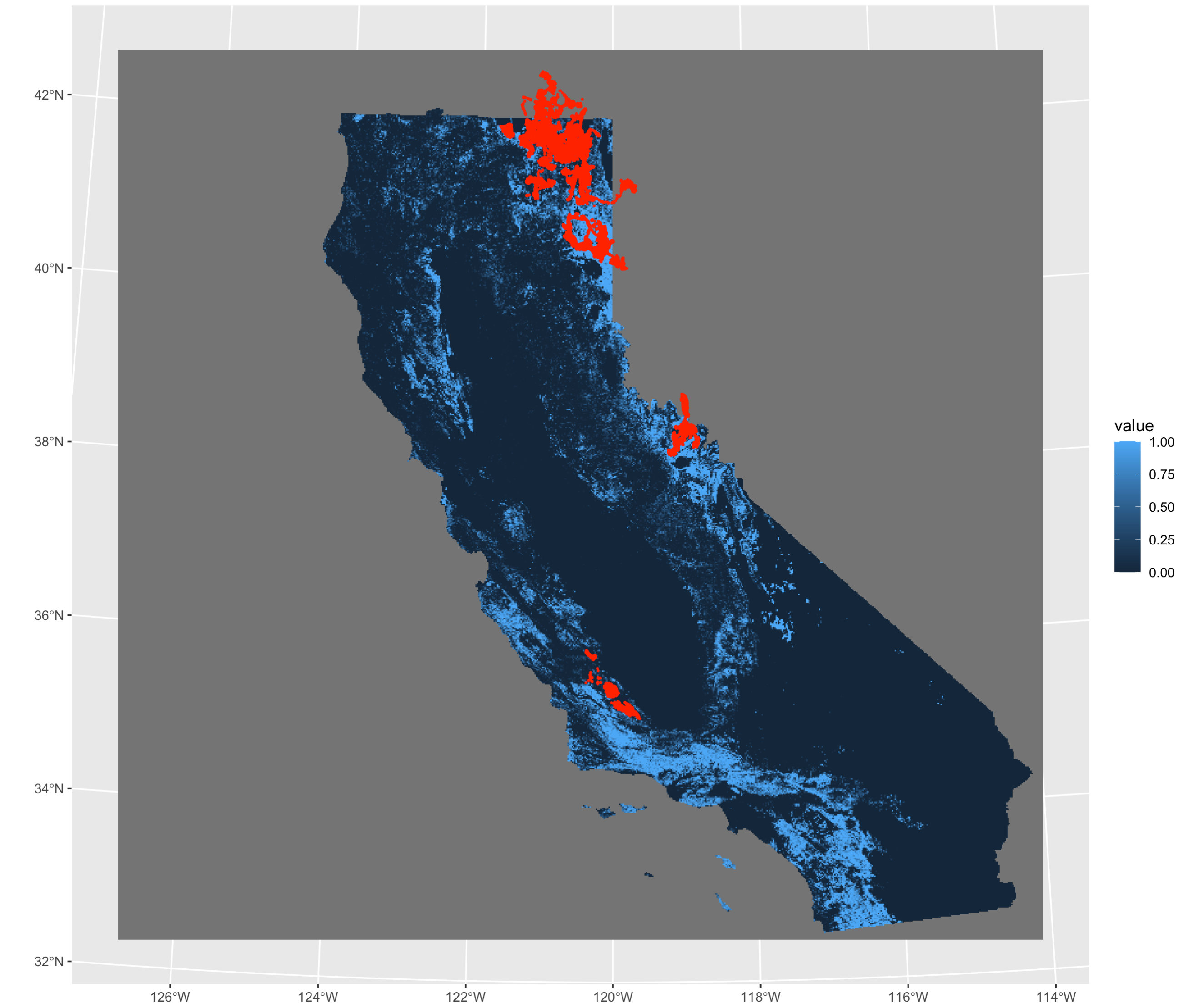


Figure 9. Pronghorn observations from GPS collars in red; proportion shrub cover in blue (calculated as the proportion of 30 m x 30 m raster cells classified as “shrub” in the CalFire FRAP vegetation dataset within a 540 m x 540 m area).


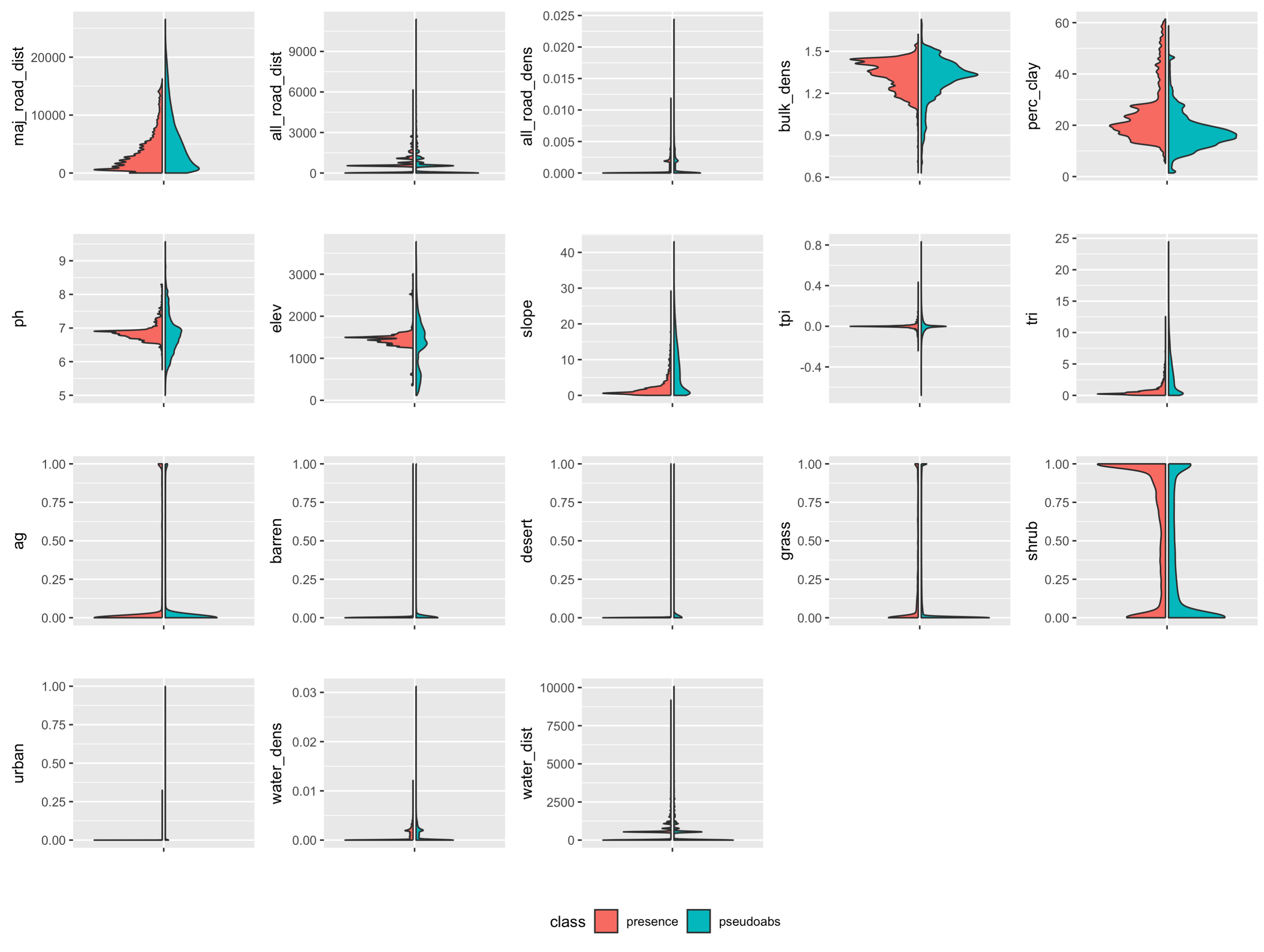


Figure 10. Distribution of predictor values at pronghorn observations collected from GPS collars distributed across three populations (red) and background points randomly generated within 25 km of occurrences. Maj_road_dist = distance to major roads (federal and state highways and county roads); all_road_dist=distance to any road in TIGER lines database; all_road_dens = density of all roads; bulk_dens = bulk density of soil; perc_clay = percentage clay in soil; ph = pH of soil; elev = elevation; slope = slope in degrees; tpi = topographic position index; tri = terrain ruggednes index; ag = proportion of cells classified as agriculture; barren = proportion of cells classified as barren; desert = proportion of cells classified as desert; grass = proportion of cells classified as grassland; shrub = proportion of cells classified as shrub; urban = proportion of cells classified as urban; water_dens = density of freshwater cover within cell;

water_dist = distance to freshwater.


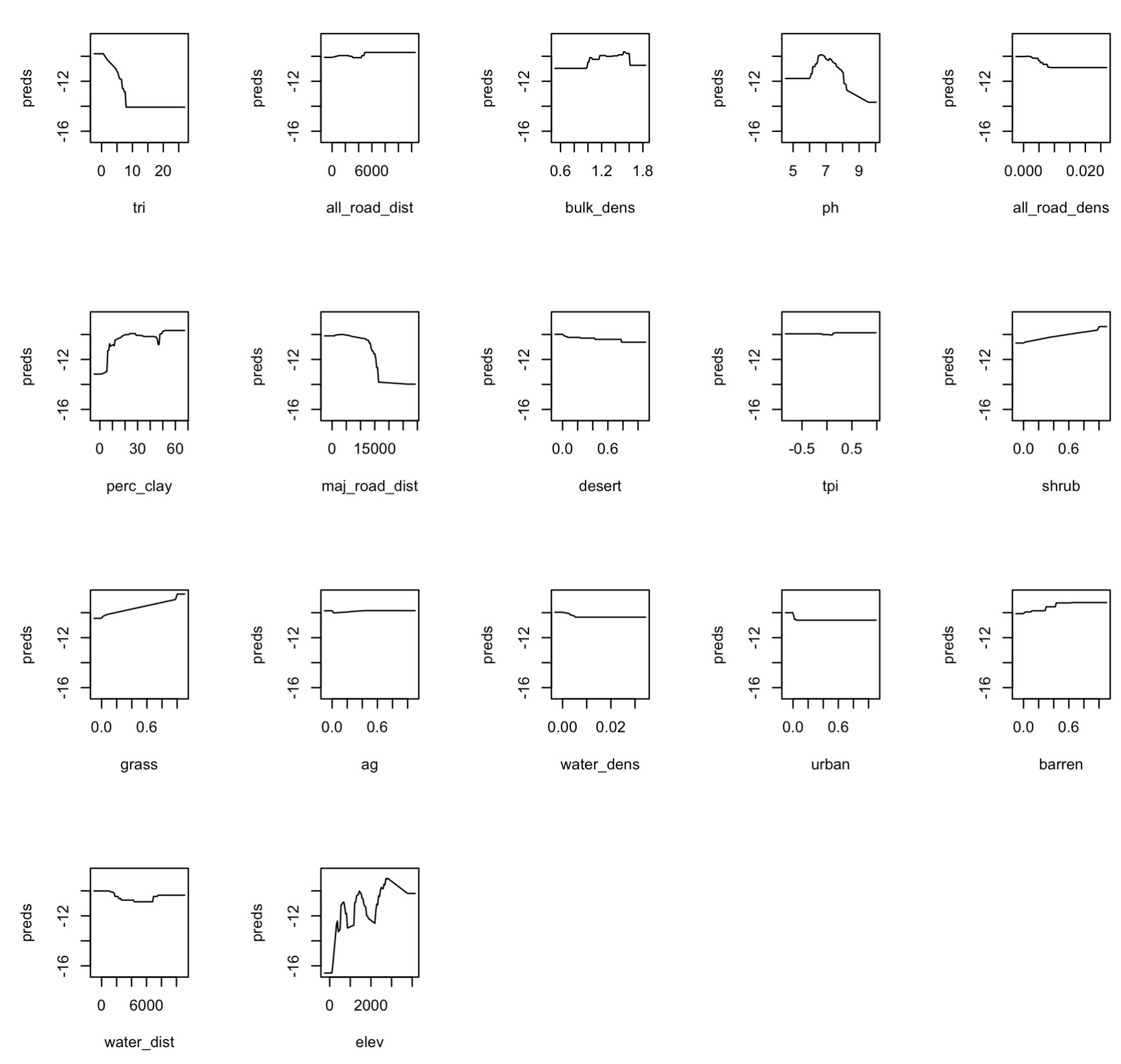


Figure 11. Response curves from a Maxent model of pronghorn habitat selection based on GPS collar data from three populations in California and the initial, full set of predictors. Tri = terrain ruggednes index; all_road_dist=distance to any road in TIGER lines database; bulk_dens = bulk density of soil; ph = pH of soil; all_road_dens = density of all roads; perc_clay = percentage clay in soil; maj_road_dist = distance to major roads (federal and state highways and county roads); desert = proportion of cells classified as desert; tpi = topographic position index; shrub = proportion of cells classified as shrub; grass = proportion of cells classified as grassland; ag = proportion of cells classified as agriculture; water_dens = density of freshwater cover within cell; urban = proportion of cells classified as urban; barren = proportion of cells classified as barren;

water_dist = distance to freshwater; elev = elevation.


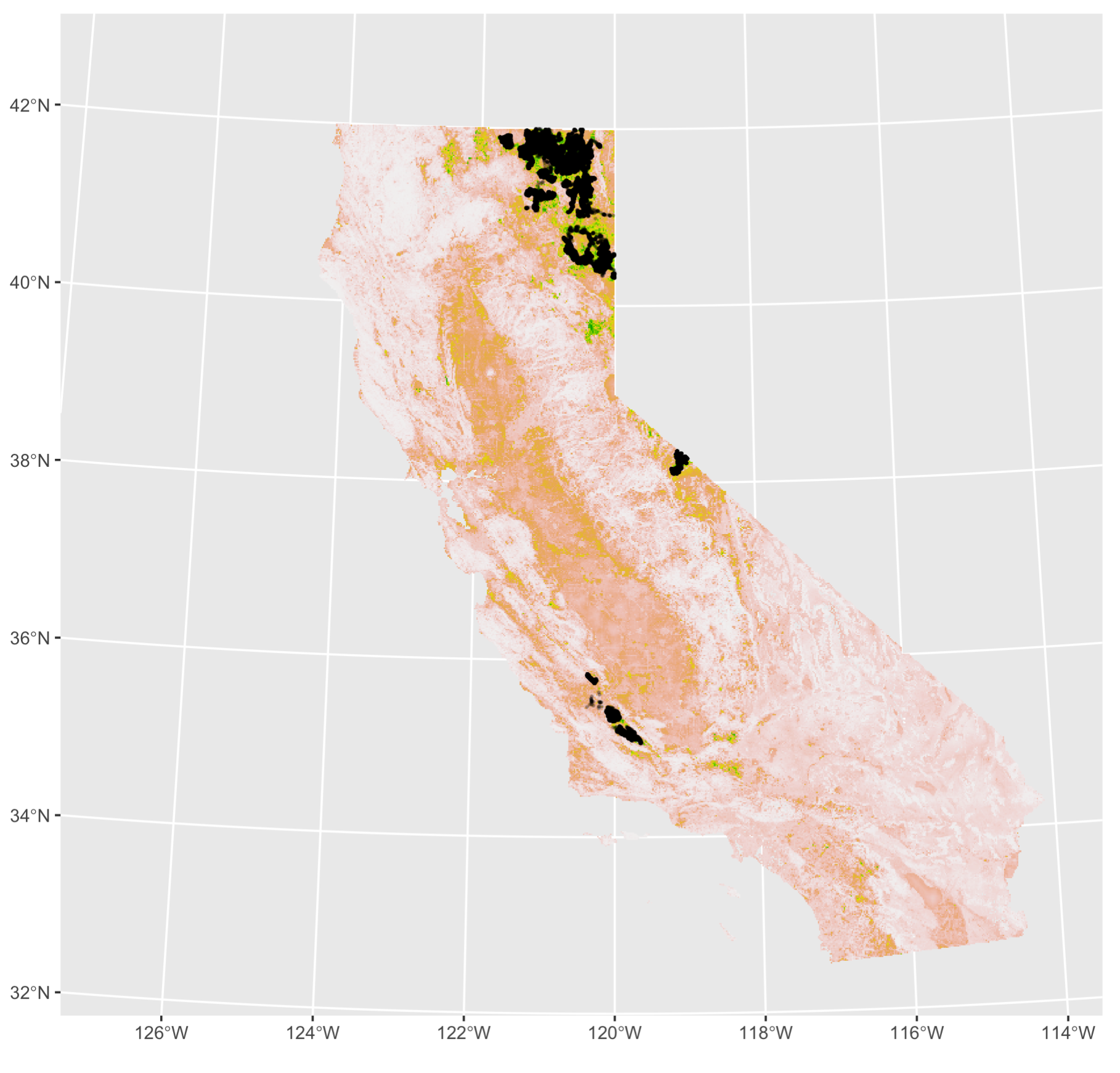


Figure 12. Predicted pronghorn habitat from ensemble of habitat suitability using Maxent, Random Forests, Classification and Regression Trees, and a Generalized Linear Model with a logistic link. Green = high suitability, white = low suitability; black points are observations collected from GPS collars on three populations of pronghorn used in model.


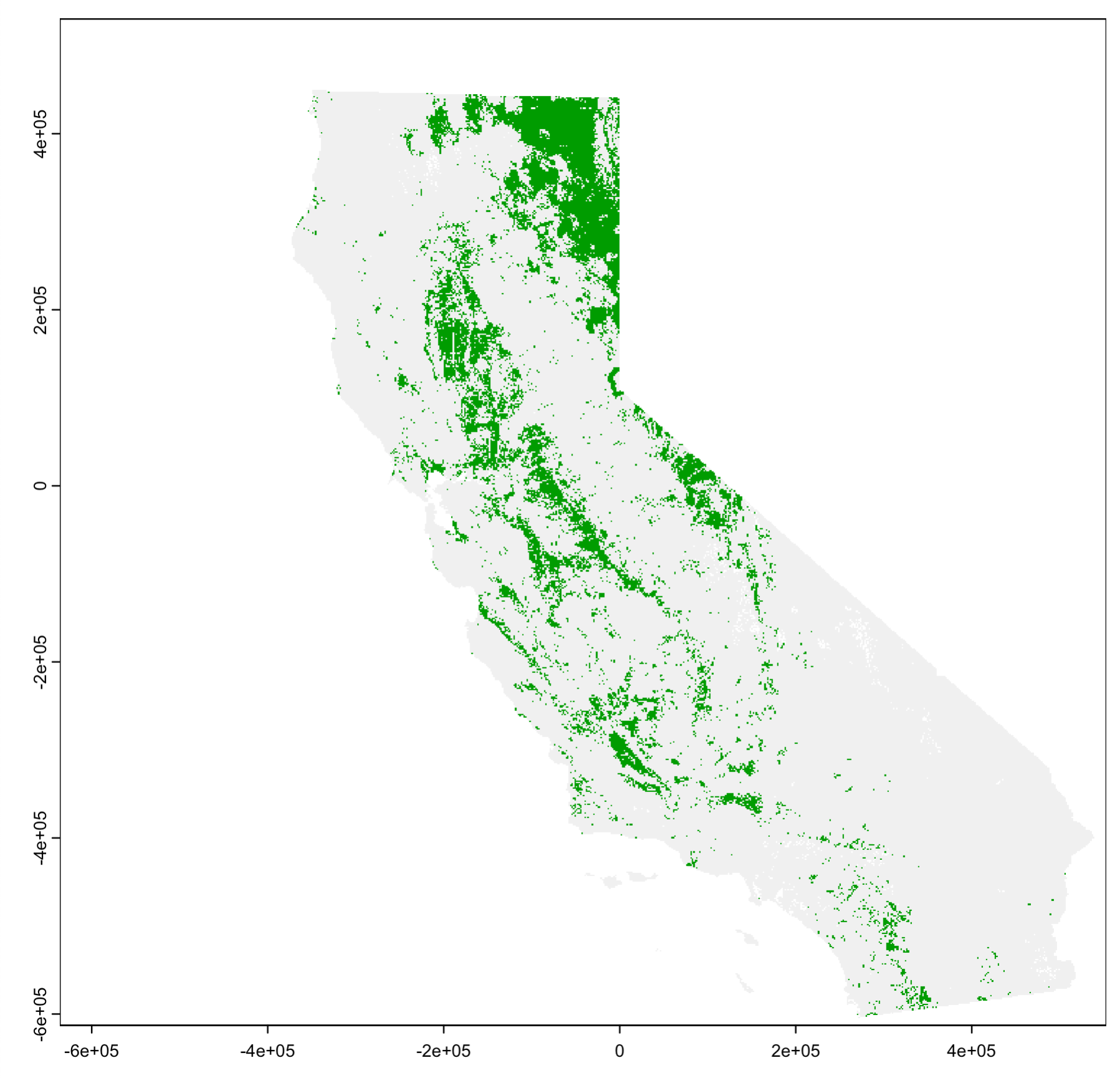


Figure 13. Ensemble model of pronghorn habitat suitability using occurrences collected from GPS collars on three populations of pronghorn; green areas represent all areas with suitability value >= any pronghorn occurrence in database used to train model.


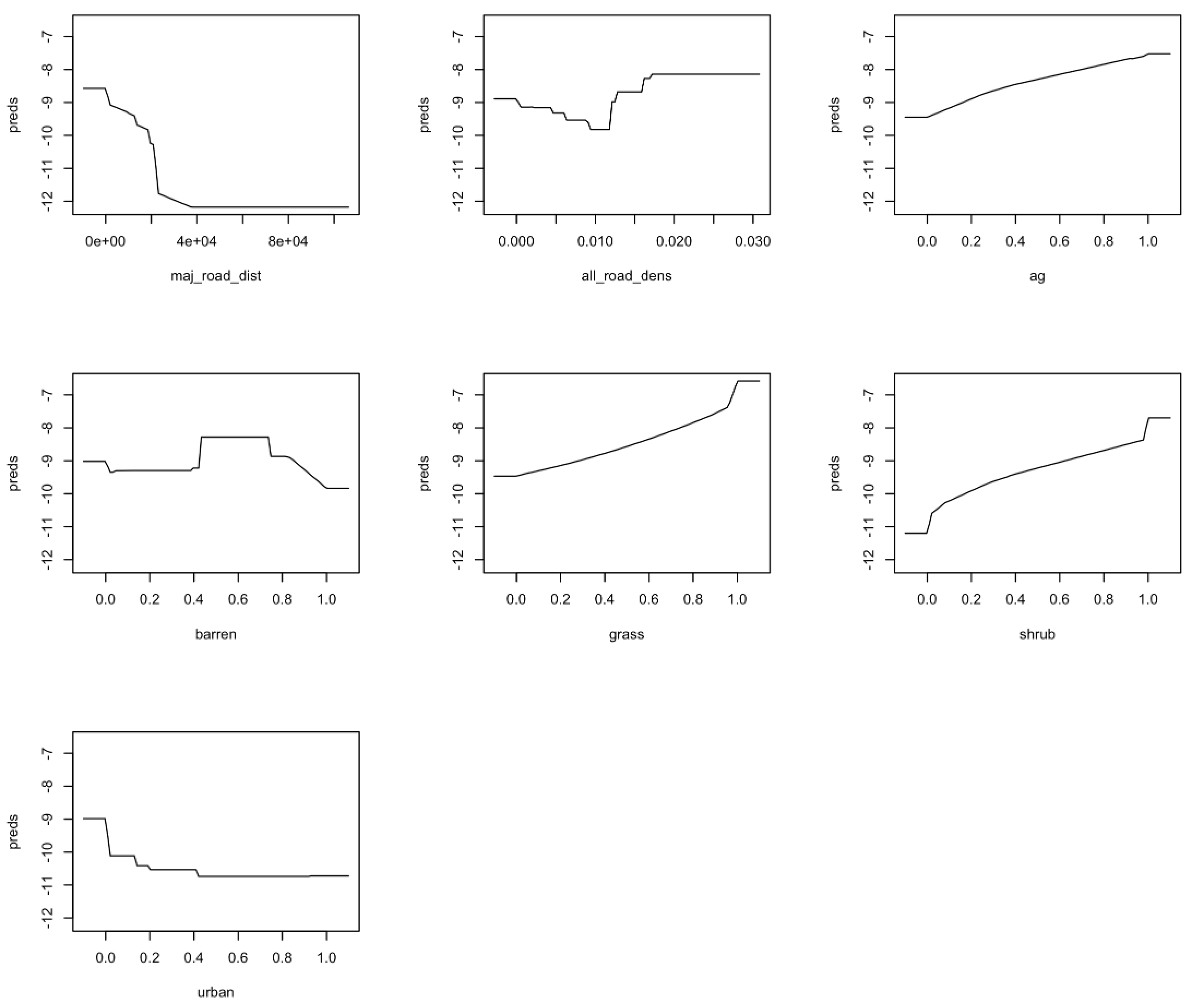


Figure 14. Response curves from a Maxent model of pronghorn habitat selection based on GPS collar data from three populations in California and a reduced set of predictors to prevent over-fitting. Maj_road_dist = distance to major roads (federal and state highways and county roads); all_road_dist=distance to any road in TIGER lines database; ag = proportion of cells classified as agriculture; barren = proportion of cells classified as barren; grass = proportion of cells classified as grassland; shrub = proportion of cells classified as shrub; urban = proportion of cells classified as urban;


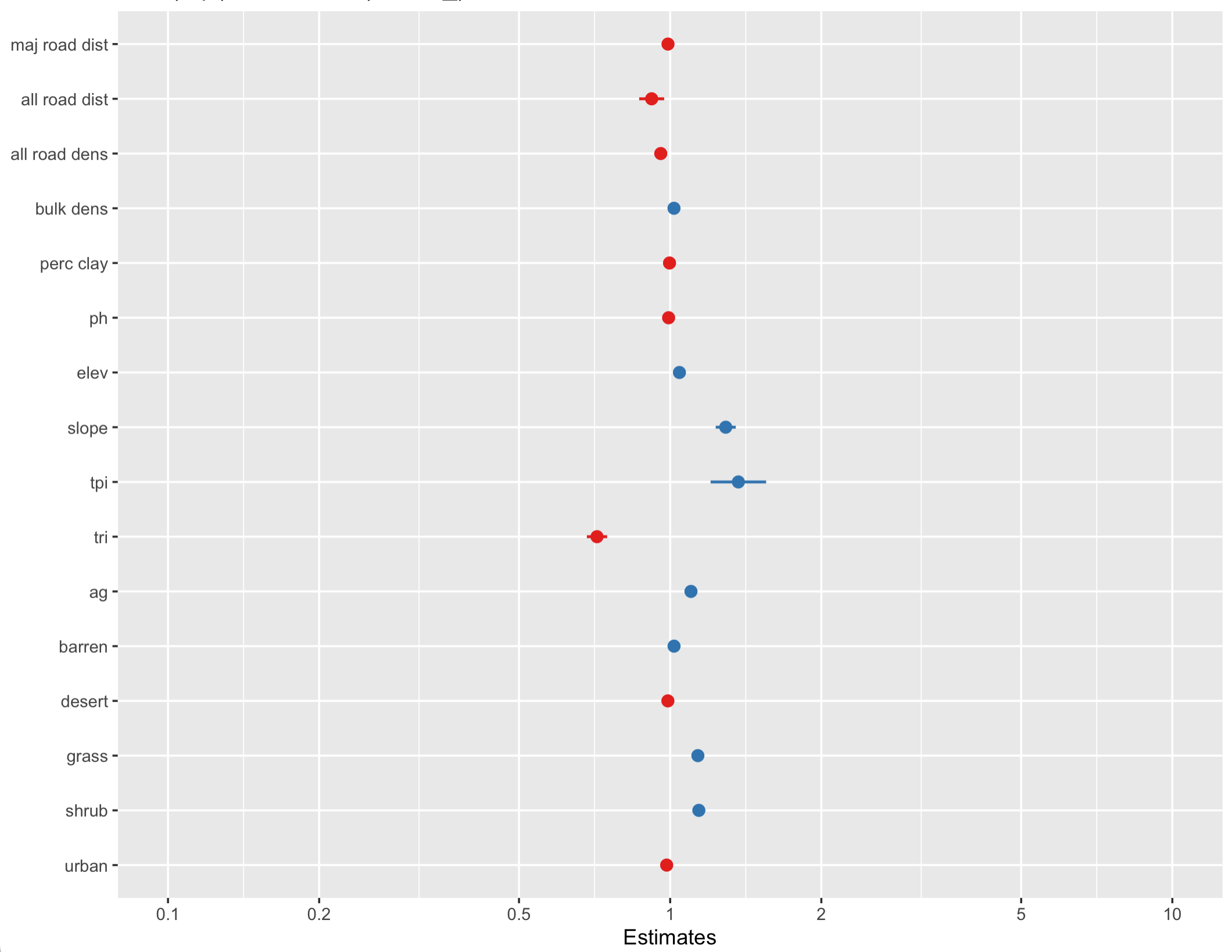


Figure 15. Standardized parameter estimates from a step selection function using GPS collar data from three populations of pronghorn in California. Maj_road_dist = distance to major roads (federal and state highways and county roads); all_road_dist=distance to any road in TIGER lines database; all_road_dens = density of all roads; bulk_dens = bulk density of soil; perc_clay = percentage clay in soil; ph = pH of soil; elev = elevation; slope = slope in degrees; tpi = topographic position index; tri = terrain ruggednes index; ag = proportion of cells classified as agriculture; barren = proportion of cells classified as barren; desert = proportion of cells classified as desert; grass = proportion of cells classified as grassland; shrub = proportion of cells classified as shrub; urban = proportion of cells classified as urban.

**
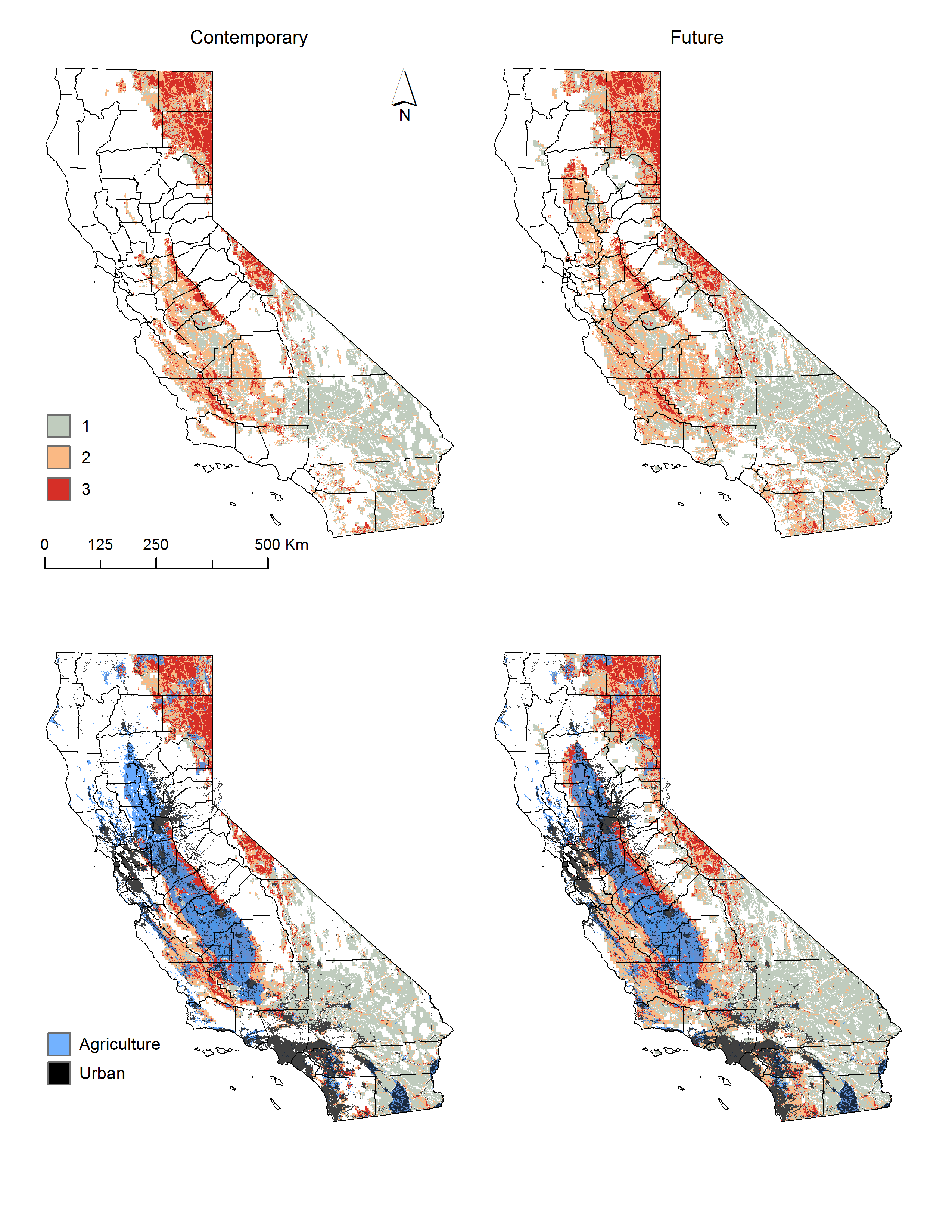
**

Figure 16. Overlay of contemporary urban and agriculture land uses with the consensus of three habitat suitability models for pronghorn using ensemble modeling, a step selection function and expert opinion; red = areas where all three models predicted pronghorn habitat; orange = two models predicted habitat; gray = one model predicted habitat.


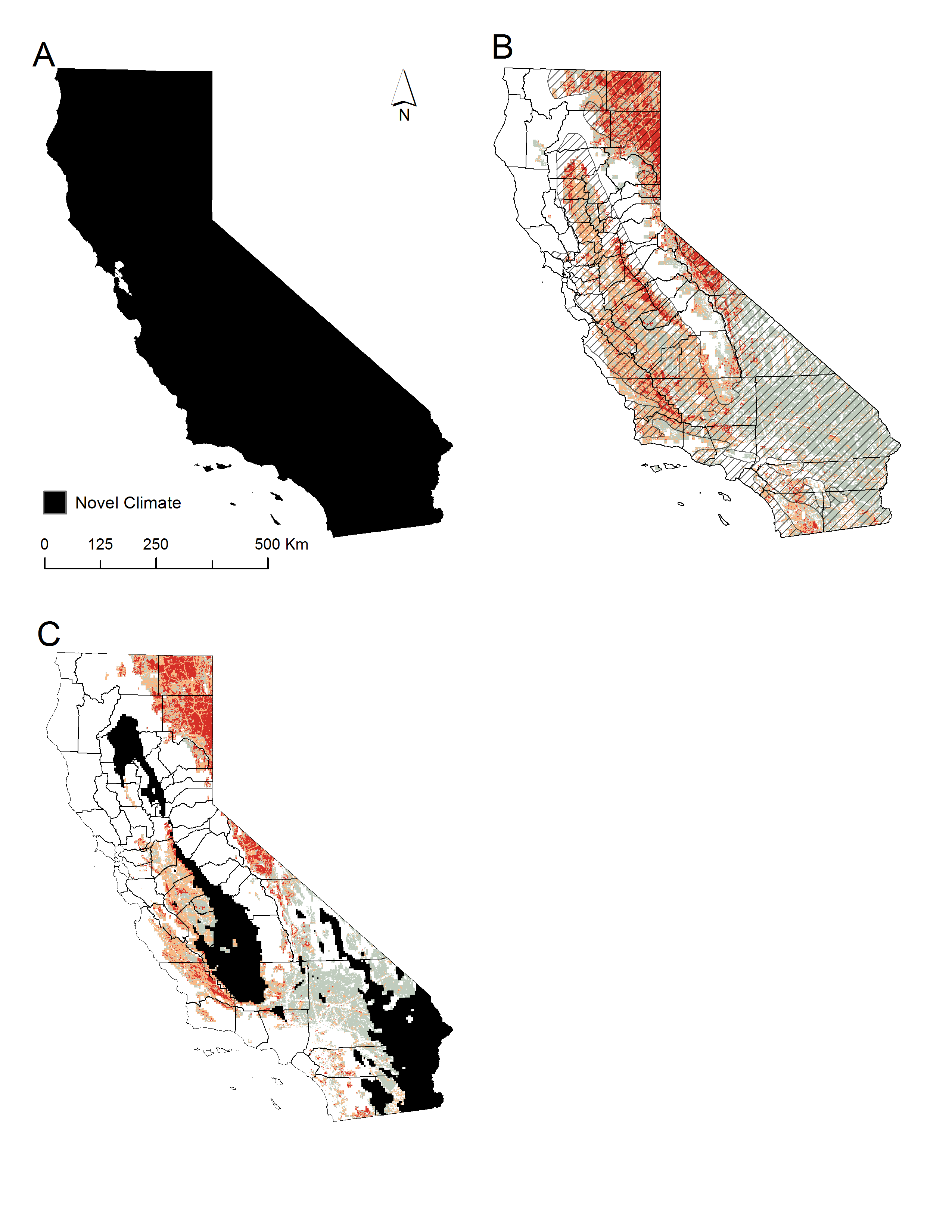


Figure 17. Areas of low similarity between contemporary and future (ACCESS CM2) climate projections for three pronghorn modeling scenarios. Black areas represent locations where the MOP metric was <0.85. A: Differences in summer maximum daily temperature and mean precipitation; B: differences in winter minimum daily temperature; C: differences between 19 contemporary and future bioclimatic variables.
