# Supplementary material for: Climatically robust multiscale species distribution models to support pronghorn recovery in California
Source: Ecol Evol. 2024 Jun 20;14(6):e11454. doi: 10.1002/ece3.11454 (PMC11188984; doi:10.1002/ece3.11454)
Supplement: Supplementary file 1 — Data S1. [file ECE3-14-e11454-s001.zip › ODMAP_Niche_reduction_Bean_EtAl_2024-01-25.docx]

Pronghorn climate distribution model - niche reduction hypothesis

– ODMAP Protocol –

2024-01-25

## Overview

#### Authorship

Contact :

<Study link>

#### Model objective

Model objective: Mapping and interpolation

Target output: Continuous habitat suitability index

#### Focal Taxon

Focal Taxon: Antilocapra americana

#### Location

Location: western North America

#### Scale of Analysis

Spatial extent: -150, -60, 15, 65 (xmin, xmax, ymin, ymax)

Spatial resolution: 1

Temporal extent: 1980-Present

Temporal resolution: 30 year mean

Boundary: buffer

#### Biodiversity data

Observation type: citizen science, standardised monitoring data, digital collection

Response data type: presence-only

#### Predictors

Predictor types: climatic, topographic

#### Hypotheses

Hypotheses: Pronghorn have undergone a niche reduction in California; distribution is driven by climate and slope

#### Assumptions

Model assumptions: Historical distribution of pronghorn in California represents contemporary climatic niche

#### Algorithms

Modelling techniques: maxent

Model complexity: used AIC to select most parsimonious model

Model averaging: none

#### Workflow

Model workflow: Used ENMEval and range of regularization multipliers / feature types to select most parsimonious model using AIC; evaluated effectiveness based on historical distribution of pronghorn in California

#### Software

Software: R 4.2.3; ENMEval; dismo; terra

Code availability: available upon request

Data availability: available upon request

## Data

#### Biodiversity data

Taxon names: Antilocapra americana

<Taxonomic reference system>

Ecological level: species

Data sources: GBIF 4/19/2023 [INSERT DOI]

Sampling design: uknown

Sample size: unknown

Scaling: only included points with certainty <500m; duplicates in climate raster were removed

Cleaning: Removed spatial outliers

<Absence data>

<Background data>

#### Data partitioning

Training data: 5-fold, checkerboard1 setting in ENMEval

<Validation data>

#### Predictor variables

Predictor variables: All Bioclim variables; slope

Data sources: Worldclim 2.1

Spatial extent: -150, -60, 15, 65 (xmin, xmax, ymin, ymax)

Spatial resolution: 1km

Coordinate reference system: EPSG = 5070

Temporal extent: 1980-2010

Temporal resolution: 30 year mean

#### Transfer data

Data sources: Future climate, all gcms WorldClim 2.1, mean output

<Spatial extent>

Spatial resolution: 1km

Temporal extent: 30 years

Temporal resolution: 30 year mean

Models and scenarios: all gcms available

<Quantification of Novelty>

## Model

#### Variable pre-selection

Variable pre-selection: Created simplest model that captured phenomenon of interest

#### Multicollinearity

<Multicollinearity>

#### Model settings

<maxent>

<Model settings (extrapolation)>

#### Model estimates

<Coefficients>

#### Model selection - model averaging - ensembles

Model selection: AIC across range of regularization multipliers, selected top model by AIC

#### Analysis and Correction of non-independence

<Spatial autocorrelation>

#### Threshold selection

Threshold selection: selection based on percentile of observations included resulting in reasonable estimate of historical distribution

## Assessment

#### Performance statistics

Performance on training data: AUC, boyce index

Performance on validation data: AIC, boyce index

<Performance on test data>

#### Plausibility check

Response shapes: Evaluated response plots

Expert judgement: Evaluated predicted range

## Prediction

#### Prediction output

<Prediction unit>

#### Uncertainty quantification

<Scenario uncertainty>

<Novel environments>
